# Remote ischemic preconditioning causes transient cell cycle arrest and renal protection by a NF-**κ**B–dependent Sema5B pathway

**DOI:** 10.1172/jci.insight.158523

**Published:** 2022-07-22

**Authors:** Jan Rossaint, Melanie Meersch, Katharina Thomas, Sina Mersmann, Martin Lehmann, Jennifer Skupski, Tobias Tekath, Peter Rosenberger, John A. Kellum, Hermann Pavenstädt, Alexander Zarbock

**Affiliations:** 1 Department of Anesthesiology, Intensive Care and Pain Medicine, University Hospital Münster, Münster, Germany.; 2 Institute of Medical Informatics, University of Münster, Münster, Germany.; 3 Department of Anesthesiology and Intensive Care Medicine, University Hospital Tübingen, Eberhard Karls University Tübingen, Tübingen, Germany.; 4 The Center for Critical Care Nephrology, Department of Critical Care Medicine, University of Pittsburgh, Pittsburgh, Pennsylvania, USA.; 5 Department of Nephrology, Internal Medicine D, University Hospital Münster, Münster, Germany.

**Keywords:** Immunology, Nephrology, Neutrophils

## Abstract

Acute kidney injury increases morbidity and mortality, and previous studies have shown that remote ischemic preconditioning (RIPC) reduces the risk of acute kidney injury after cardiac surgery. RIPC increases urinary high mobility group box protein-1 (HMGB1) levels in patients, and this correlates with kidney protection. Here, we show that RIPC reduces renal ischemia-reperfusion injury and improves kidney function in mice. Mechanistically, RIPC increases HMGB1 levels in the plasma and urine, and HMGB1 binds to TLR4 on renal tubular epithelial cells, inducing transcriptomic modulation of renal tubular epithelial cells and providing renal protection, whereas TLR4 activation on nonrenal cells was shown to contribute to renal injury. This protection is mediated by activation of induction of AMPKα and NF-κB; this induction contributes to the upregulation of Sema5b, which triggers a transient, protective G_1_ cell cycle arrest. In cardiac surgery patients at high risk for postoperative acute kidney injury, increased HMGB1 and Sema5b levels after RIPC were associated with renal protection after surgery. The results may help to develop future clinical treatment options for acute kidney injury.

## Graphical abstract

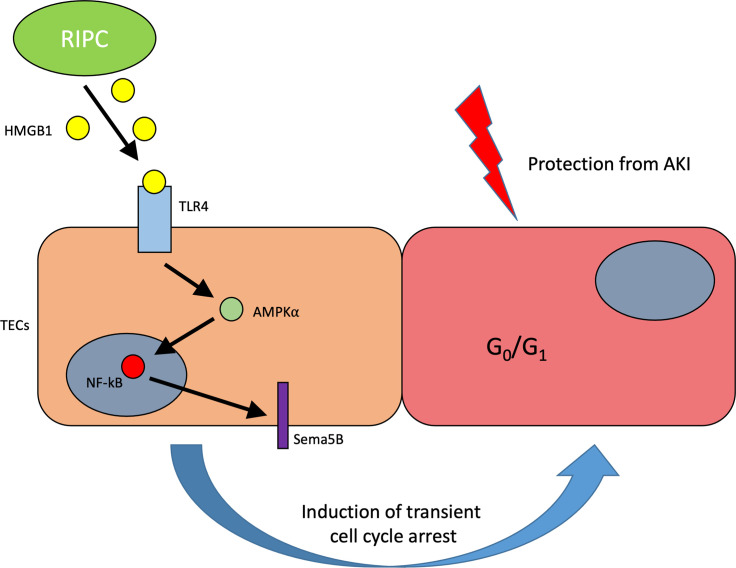

## Introduction

Acute kidney injury (AKI) is a frequent complication of critical illness and of major surgery, and it causes considerable harm (1, 2). The rates of AKI among critically ill patients can be as high as 70%, with an in-hospital mortality as high as 50% when AKI is part of the multiple organ dysfunction syndrome (3). Despite progress in therapeutic strategies, the mortality of patients after AKI remains very high (4). The mechanisms underlying the pathogenesis of AKI are complex and include inflammation, apoptosis and necrosis, mitochondrial dysfunction, reactive oxygen species (ROS), and endoplasmic reticulum (ER) stress (5–9). To date, there are no approved drug therapies for patients with AKI.

Recent studies have shown that renal cell cycle arrest is present during AKI (10). Research into the pathogenesis of AKI has demonstrated that cell cycle arrest plays an important role in self-protection and adaptive repair of tubular epithelial cells (11, 12). The cell may use the cell cycle arrest as a protective mechanism to prevent cell division when potentially damaged (13). However, if the cells do not restart the cell cycle and remain in cell cycle arrest, a fibrotic phenotype can ensue. Tissue inhibitor of metalloproteinases-2 (TIMP-2) and insulin-like growth factor-binding protein 7 (IGFBP7) are known to be involved in the G_1_ cell cycle arrest phase noted to occur during the very early phases of cellular stress (14–16). It has been demonstrated that renal tubular epithelial cells also go through this G_1_ cell cycle arrest phase following stress that can be caused by different insults (17). G_1_ cell cycle arrest is reversible; however, persistent cell cycle arrest may serve as a mechanistic link between AKI and CKD since sustained cell cycle arrest will result in a senescent cell phenotype and lead to fibrosis (18).

Remote ischemic preconditioning (RIPC), defined as transient brief episodes of ischemia at a remote site before a subsequent injury of the target organ, can trigger adaptive responses that protect against various insults. It has been hypothesized that RIPC includes systemic multifactorial neuronal, humoral, and antiinflammatory signaling pathways, which may vary in response to different ischemic stimuli and are likely to interact with each other (19). Several clinical studies have shown beneficial effects of RIPC on different organs, including the heart (20–23), kidney (19, 24–26), lung (27), and brain (28). Experimental and clinical evidence indicates that RIPC might be an effective measure to protect kidneys from injury, at least in certain high-risk patients. If effective, RIPC could offer an inexpensive, novel, and noninvasive strategy to reduce the occurrence of AKI in different clinical scenarios (24, 26). However, large RCTs in unselected patients have been negative, and there is a need to better understand the mechanisms of RIPC protection so that it can be used appropriately (29, 30). In a clinical study of patients undergoing cardiac surgery, we demonstrated that high mobility group box protein-1 (HMGB1) appeared in the urine in response to RIPC and was associated with the expression of the 2 cell cycle arrest markers TIMP-2 and IGFBP7 (24). Furthermore, protection from AKI with RIPC was only observed in patients exhibiting an increase in TIMP-2 and IGFBP7 (31).

We hypothesize that RIPC induces the release of HMGB1 that subsequently activates natural defenses such as temporary cell cycle arrest. These defenses, once engaged, can then protect the kidney during subsequent inflammatory, toxic, or ischemic stress. In this study, using in vitro assays, a renal ischemia-reperfusion injury (IRI) model, and samples from patients undergoing cardiac surgery, we investigate whether RIPC can protect against renal IRI and whether this protection is HMGB1 dependent.

## Results

### RIPC protects the murine kidney from IRI by HMGB1 signaling in vivo.

**Figure 1:**
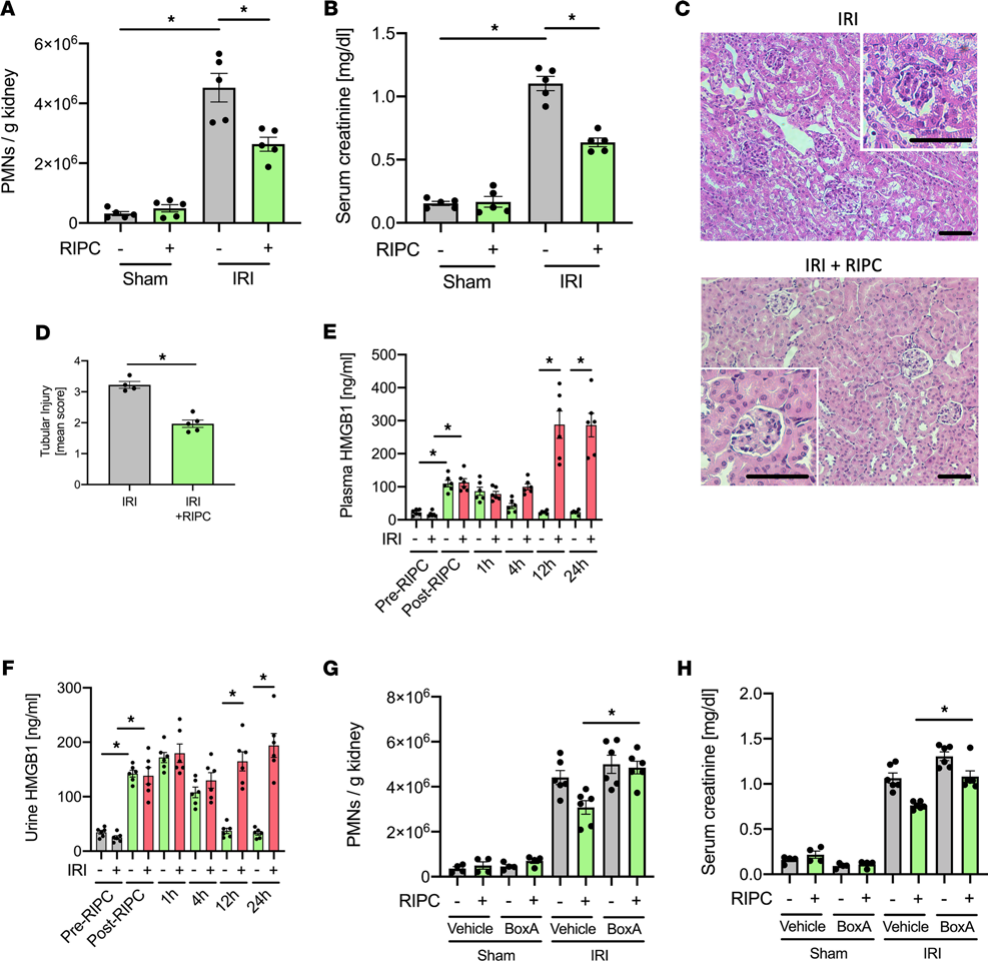
Remote ischemic preconditioning (RIPC) protects the murine kidney from IRI by HMGB1 signaling in vivo. After induction of general anesthesia WT mice received either 3 cycles of 5-minute RIPC by inflation of a blood pressure cuff (200 mmHg) positioned to upper leg of the hind limb interrupted by 5-minute reperfusion intervals following cuff deflation. In the control group, the cuff was inflated to 20 mmHg, not resulting in limb ischemia. IRI was induced in WT mice by clamping of the renal pedicles for 32 minutes. Twenty-four hours after the surgery, mice were sacrificed. (**A**) The recruitment of neutrophils (PMNs) into the kidney was analyzed by flow cytometry (*n* = 5). (**B**) Serum creatinine levels were measured by a photometric assay (*n* = 5). (**C** and **D**) Exemplary histological images and quantification of histological tubular injury (*n* = 4–5). Scale bar: 100 µm. (**E** and **F**) Plasma HMGB1 levels and urinary HMGB1 levels were analyzed before and after RIPC or control procedure (*n* = 6). Some mice received either BoxA or a vehicle control before inducing RIPC prior to renal IRI. Twenty-four hours after IRI, mice were sacrificed. (**G**) The recruitment of neutrophils (PMNs) into the kidney was analyzed by flow cytometry (*n* = 4-6). (**H**) Serum creatinine levels were measured by a photometric assay (*n* = 4-6). Mann-Whitney *U* test (**D**) and 1-way ANOVA followed by Bonferroni testing (**A**, **B**, and **E**–**H**) were used for statistical analysis; *P < 0.05.

RIPC before inducing renal IRI significantly reduced AKI severity, as shown by reduced neutrophil recruitment into the kidney, decreased serum creatinine levels, histological renal tubular injury, and histological evidence for neutrophil infiltration (Figure 1, A–D, and Supplemental Figure 1, A and B; supplemental material available online with this article; https://doi.org/10.1172/jci.insight.158523DS1). In addition, RIPC increased plasma and urine HMGB1 concentrations (Figure 1, E and F). Interestingly, RIPC before IRI-induced AKI also led to increased plasma and urine HMGB1 concentration at later time points (Figure 1, E and F). RIPC efficacy was similar when the blood pressure cuff was placed around the upper leg of the front limb instead of the hind limb (Supplemental Figure 1, C–F). To further characterize the role of HMGB1 in RIPC-induced protection from AKI, we used a specific inhibitor (BoxA, 300 μg/mouse i.p.), which represents an inactive part of the HMGB1 molecule and blocks ligand binding of endogenous HMGB1 in vivo (32). Blocking the binding of HMGB1 before inducing RIPC and renal IRI significantly abolished the renoprotective effects of RIPC (Figure 1, G and H).

### RIPC induces differential transcriptomic reprogramming in murine neutrophils and renal tubular epithelial cells.

**Figure 2:**
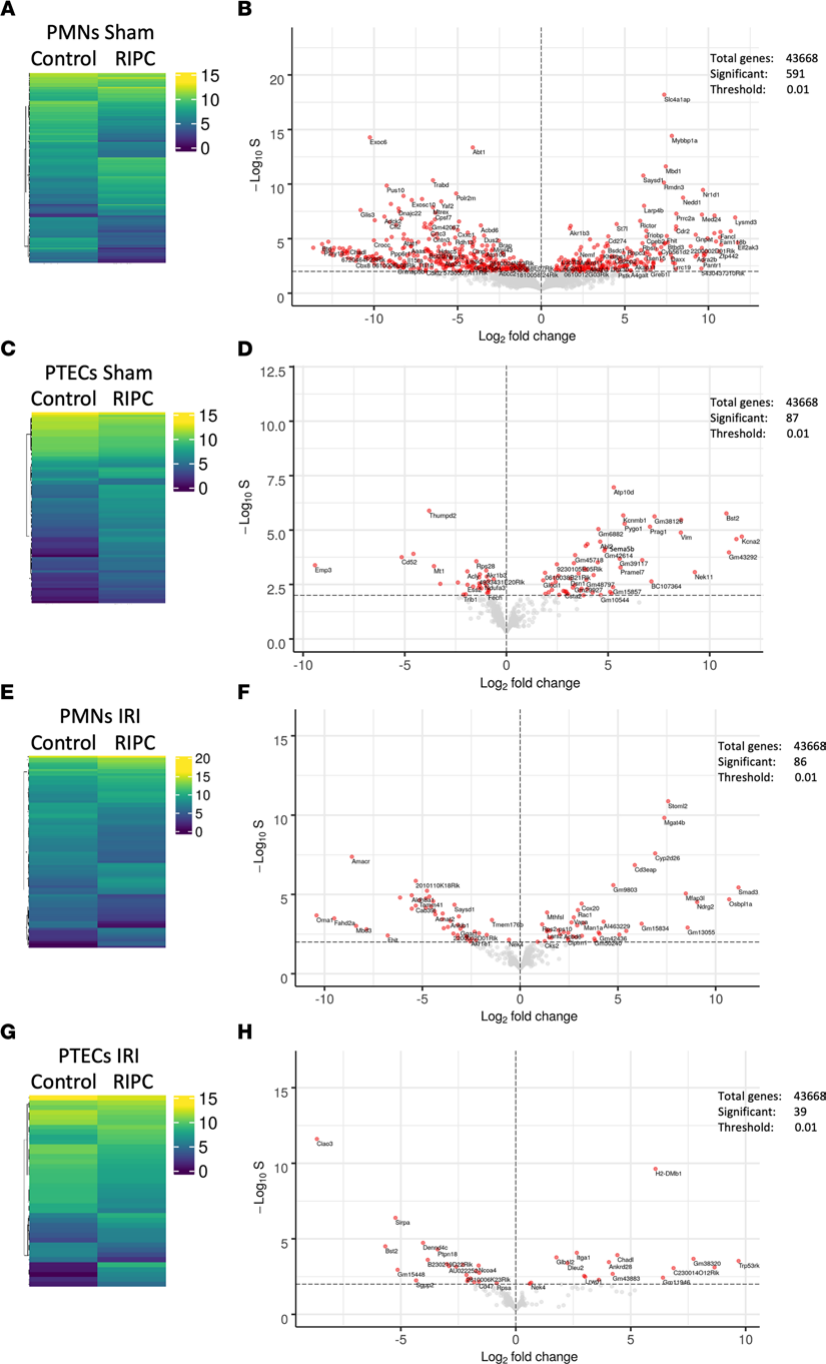
RIPC induces differential transcriptomic reprogramming in murine neutrophils and renal tubular epithelial cells. After induction application of RIPC or control procedure, IRI was induced in WT mice by clamping of the renal pedicles for 32 minutes. Twenty-four hours after the surgery, mice were sacrificed. In order to conduct RNA-Seq, neutrophils and proximal renal tubular epithelial cells (PTECs) were isolated 24 hours after the IRI procedure (*n* = 3 biological replicate libraries per group). (**A**–**H**) Heatmaps with hierarchical tree and volcano plots comparing differences of RNA-Seq–based gene expression values in PMNs from sham mice (**A** and **B**) and IRI mice (**E** and **F**), as well as from PTECs from sham mice (**C** and **D**) and IRI mice (**G** and **H**). Biostatistical analysis described in Methods; *P <0.05.

One putative target receptor for HMGB1 among other receptors (e.g., TLR5, RAGE, and CXCR4), TLR4, is expressed on both neutrophils and renal tubular epithelial cells. Under baseline conditions, the application of RIPC led to the differential regulation of a total of 591 genes in neutrophils and 87 genes in renal tubular epithelial cells (Figure 2, A–D). In animals receiving RIPC before IRI, 86 genes in neutrophils and 39 genes in renal tubular epithelial cells (Figure 2, E–H) were differentially regulated compared with IRI without RIPC. In polymorphonuclear neutrophils (PMNs) isolated from animals receiving RIPC before IRI, we found differential expression of genes governing nuclear signaling (*Mfap3L*), cell proliferation, differentiation, and cell death (*Smad3*); physiological plasticity; and cellular stress response (*Oma1*). In renal tubular epithelial cells, genes that modulate nuclear transcription factor activity (e.g., *Trp53rkb*, *Ankrd28*) and molecules involved in pattern recognition (*H2DMb1*) were significantly upregulated.

### HMGB1 acts by binding to TLR4 on murine renal tubular epithelial cells.

**Figure 3:**
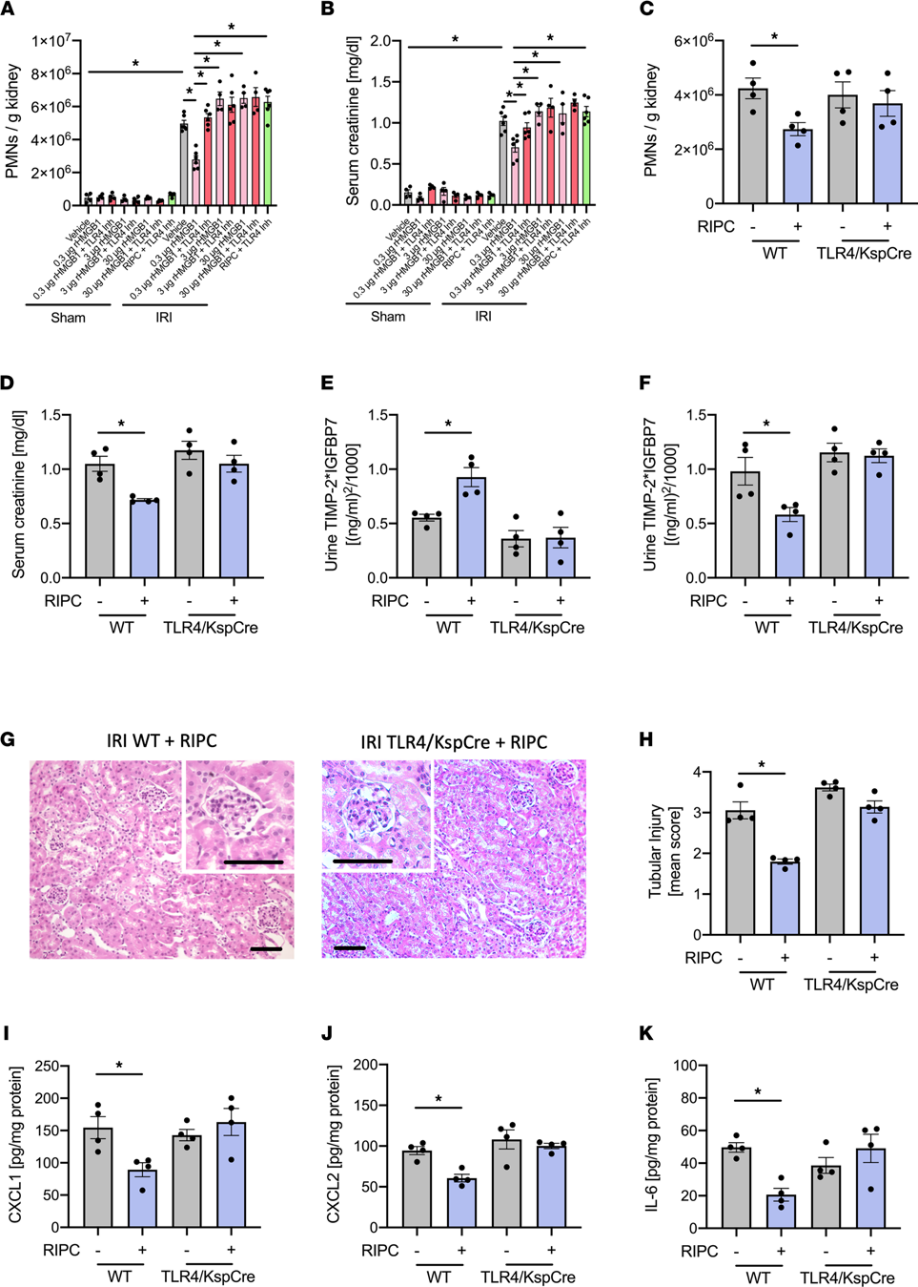
Administration of HMGB1 protects from AKI, and blocking HMGB1 abrogates the protective effect of RIPC. After induction of general anesthesia, renal IRI was induced in WT mice by clamping of the renal pedicles for 32 minutes. Some groups received rHMGB1 (0.3, 3, or 30g/mouse) or rHMGB1 plus a TLR4 inhibitor before the induction of renal IRI. Twenty-four hours after the surgery, mice were sacrificed. (**A**) The recruitment of neutrophils (PMNs) into the kidney was analyzed by flow cytometry (*n* = 4–6). (**B**) Serum creatinine levels were measured by a photometric assay (*n* = 4–6). In additional experiments, IRI was induced in WT control and TLR4^fl/fl^/Ksp-Cre^+/T^ mice, which did or did not receive RIPC prior to IRI induction by clamping of the renal pedicles for 32 minutes. Twenty-four hours after the surgery, mice were sacrificed. (**C**) The recruitment of neutrophils (PMNs) into the kidney was analyzed by flow cytometry (*n* = 4). (**D**) Serum creatinine levels were measured by a photometric assay (*n* = 4). (**E** and **F**) The biomarkers TIMP-2 and IGFBP7 were measured in urine samples after RIPC application (**E**) and 24 hours after IRI (**F**). (**G** and **H**) Exemplary histological images and quantification of histological tubular injury (*n* = 4). Scale bar: 100 µm. (**I**–**K**) The levels of the chemokines CXCL1 (**I**), CXCL2 (**J**), and IL-6 (**K**) in kidney tissue homogenisates were analyzed by ELISAs (*n* = 4). One-way ANOVA followed by Bonferroni testing was used for statistical analysis; *P < 0.05.

The administration of recombinant HMBG1 (rHMGB1) at a low concentration of 0.3 μg/mouse before inducing renal IRI significantly attenuated both neutrophil recruitment and serum creatinine increase (Figure 3, A and B). This protective effect was completely reversed when TLR4 was pharmacologically blocked by TAK-242 (3 mg/kg i.p.), which blocks the HMGB1 binding site on TLR4 before renal IRI was applied (Figure 3, A and B) (33). Likewise, blocking TLR4 also completely abrogated the protective effects of RIPC (Figure 3, A and B). Interestingly, the administration of rHMGB1 at higher concentrations of 3 and 30 μg/mouse injected i.v. before inducing renal IRI significantly increased both neutrophil recruitment and serum creatinine levels, and these effects were reversed by simultaneous administration of TAK-242 (3 mg/kg i.p.) — suggesting that lower HMGB1 levels have a protective effect, whereas higher levels induce inflammation and reduced kidney function (Figure 3, A and B). Furthermore, the protective effect of RIPC on the development of IRI-induced AKI was also completely abrogated in conditional TLR4-KO mice specifically lacking TLR4 on renal tubular epithelial cells, where the cell-specific deletion of TLR4 was achieved by using Ksp-Cre mice, allowing us to selectively target proteins in proximal tubules for genetic ablation (TLR4^fl/fl^Ksp-Cre^+/T^, or TLR4/Ksp-Cre; Figure 3, C and D). RIPC induced the increase of the combined product of the biomarkers ([TIMP-2]*[IGFBP7]) in the urine of WT mice before inducing renal IRI, but it did not do so in TLR4/Ksp-Cre mice (Figure 3E and Supplemental Figure 7). Likewise, applying RIPC in WT mice led to decreased urinary concentrations of [TIMP-2]*[IGFBP7] 24 hours after IRI, indicative of reduced IRI-induced renal tubular injury (Figure 3, F–H, and Supplemental Figure 7). These protective effects of RIPC were absent in TLR4/Ksp-Cre mice (Figure 3, F–H, and Supplemental Figure 7). The cytokine levels of all chemokines in the homogenate of the kidneys were significantly decreased in WT mice receiving RIPC before inducing renal IRI compared with WT, which did not receive RIPC before inducing renal IRI. However, this was not the case in TLR4/Ksp-Cre mice, suggesting that RIPC reduces the cytokine production in the kidney and subsequently reduces PMN recruitment into the kidneys (Figure 3, I–K).

To demonstrate that the protective effect of RIPC is specifically conveyed by TLR4, we performed IRI with or without prior RIPC in WT and TLR5-KO mice and showed that genetic deletion of TLR5 does not interfere with the protective effect of RIPC (Supplemental Figure 2, A–E). These data support our conclusion that release of HMGB1 from the ischemic tissue elicited by the RIPC procedure causes direct protection from AKI at the renal tubular epithelial cell level in a TLR4-dependent fashion. Interestingly, TLR4 has been shown to be involved in the pathogenesis of IRI-induced AKI, as mice with a global KO of TLR4 were protected from AKI (34). Indeed, the administration of the pharmacological TLR4 blocker TAK-242 before the induction of IRI protects mice from IRI-induced AKI (Supplemental Figure 2, F and G). Interestingly, if mice were neutrophil depleted by using a depleting antibody (clone RB6-8C5, 30 mg/mouse i.p. 24 hours before the experiment) and reconstituted with donor neutrophils isolated form the BM of WT mice, incubation of the donor neutrophils with the TLR4 inhibitor TAK-242 prior to injection into the recipient mice protected the recipient from AKI elicited by subsequent IRI surgery compared with mice that were reconstituted with vehicle-treated neutrophils (Supplemental Figure 2, H and I). Thus, TLR4 on cells other than renal proximal renal tubular epithelial cells (PTECs) (as targeted in our conditional TLR4^fl/fl^ Ksp-Cre mice) such as neutrophils may be the main driver of IRI-induced renal damage.

### The TLR4-dependent signaling triggered by HMGB1 protects the kidney from IRI in vivo in the murine system.

**Figure 4:**
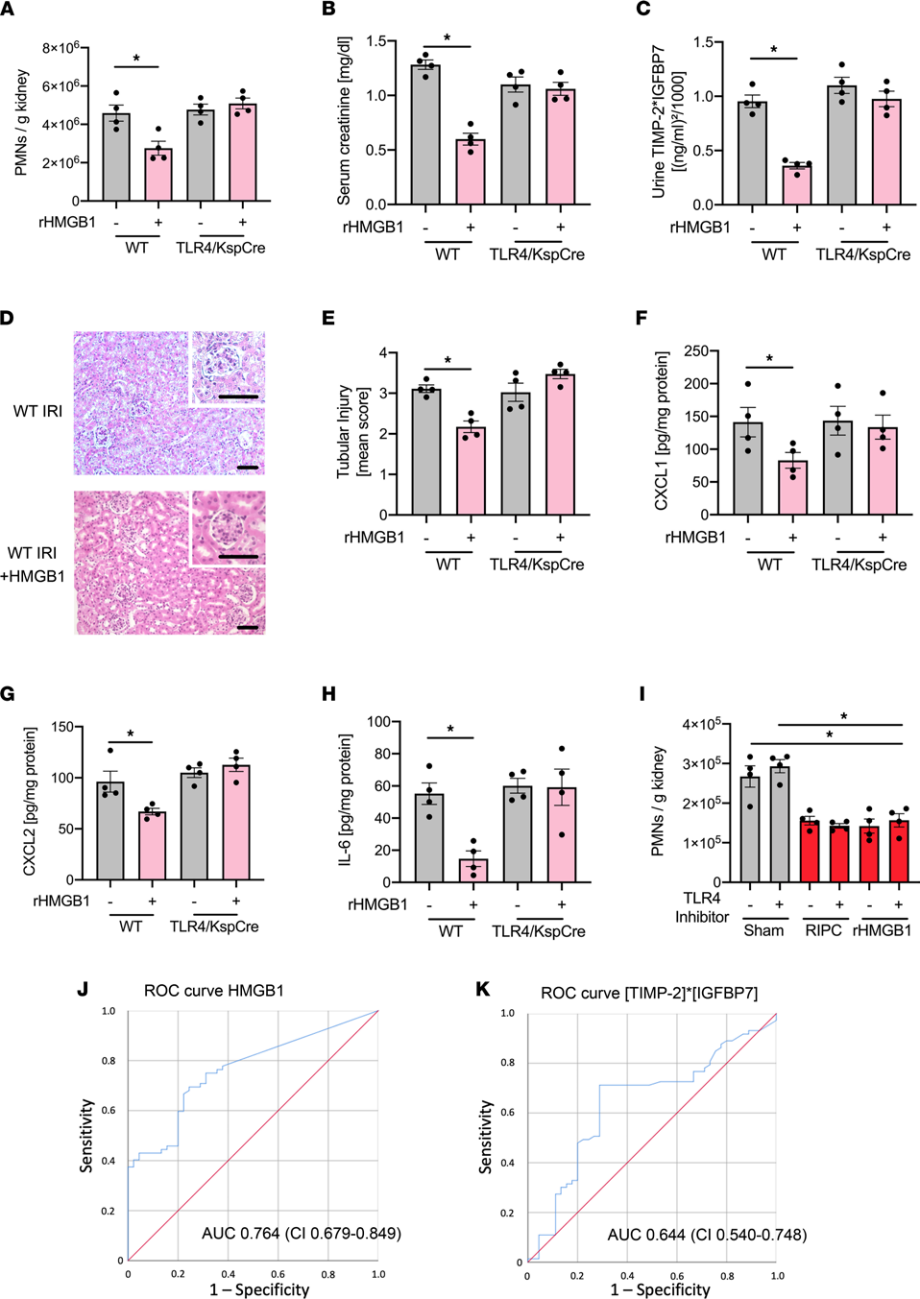
The TLR4-dependent signaling triggered by HMGB1 protects the kidney from ischemia-reperfusion injury in vivo in the murine system and in humans. After induction of general anesthesia, renal IRI was induced in WT mice by clamping of the renal pedicles for 32 minutes. Some WT and TLR4^fl/fl^/Ksp-Cre^+/T^ mice received HMGB1 prior to IRI procedure. Twenty-four hours after the surgery, mice were sacrificed. (**A**) The recruitment of neutrophils (PMNs) into the kidney was analyzed by flow cytometry (*n* = 4). (**B**) Serum creatinine levels were measured by a photometric assay (*n* = 4). (**C**) The biomarkers TIMP-2 and IGFBP7 were measured in urine samples 24 hours after renal IRI (*n* = 4). (**D** and **E**) Exemplary histological images and quantification of histological tubular injury (*n* = 4). Scale bar: 100 µm. (**F**–**H**) The levels of the chemokines CXCL1 (**F**), CXCL2 (**G**), and IL-6 (**H**) in kidney tissue homogenisates was analyzed by ELISAs (*n* = 4). WT Neutrophils were isolated and incubated with a pharmacological TLR4 receptor blocker or vehicle control, reinjected into WT recipient mice in a 1:1 ratio, and mice were subjected to RIPC or control procedure or were injected with rHMGB1 (0.3 μg/mouse i.v.) or vehicle control procedure before IRI induction. (**I**) The recruitment of neutrophils (PMNs) into the kidney was analyzed by flow cytometry (*n* = 4). Receiver operating characteristic analyses in patients receiving RIPC. (**J**) AUC for HMGB1. (**K**) AUC for [TIMP-2]*[IGFBP7]. One-way ANOVA followed by Bonferroni testing was used for statistical analysis; *P < 0.05.

The administration of HMGB1 to WT mice also ameliorated IRI-induced AKI — as shown by reduced neutrophil recruitment into the kidney and reduced serum creatinine levels — which was abolished in TLR4/Ksp-Cre mice, indicating that the specific binding of HMGB1 to TLR4 on renal tubular epithelial cells is required for the protection from IRI-induced AKI in vivo (Figure 4, A and B). Likewise, the administration of HMGB1 before inducing renal IRI led to decreased urinary concentrations of the renal injury biomarkers TIMP-2 and IGFBP7, reduced renal tissue injury 24 hours after IRI, and was absent in TLR4/Ksp-Cre mice (Figure 4, C–E, and Supplemental Figure 7). Other than in WT mice, HMGB1 did not reduce the cytokine levels in the kidneys of TLR4/Ksp-Cre mice (Figure 4, F–H). To exclude that RIPC-induced release of HMGB1 or the administration of HMGB1 at a low dose has a direct effect on neutrophil recruitment, we performed a competitive neutrophil recruitment assay into the kidney. For this purpose, we isolated neutrophils from WT donor mice and incubated the cells with a pharmacological TLR4 receptor blocker or vehicle control and different cell dyes. The cells were reinjected into WT recipient mice in a 1:1 ratio, and mice were subjected to RIPC or control procedure or received rHGMB1 (0.3 μg/mouse) or vehicle control before IRI induction. RIPC did not affect neutrophil recruitment of both neutrophil populations, suggesting that RIPC-induced release of HMGB1 does not directly affect neutrophil recruitment (Figure 4I).

### HMGB1 levels after RIPC are predictive for improved renal function in human patients after cardiac surgery.

We analyzed the urinary concentrations of HMGB1 after RIPC application in a cohort of 240 cardiac surgery patients included in the RenalRIP trial. Baseline demographic data do not differ between the patient groups (24). In total, 62.5% (75 of 120) of patients did not develop AKI within 72 hours after surgery. We observed that an early increase in urinary HMGB1 concentration predicted protection from AKI (Figure 4J). The predictive value was even higher than the early [TIMP-2]*[IGFBP7] increase after RIPC application (Figure 4K). The cutoff value of post-HMGB1 (i.e., the urine HMGB1 level after application of RIPC) in the RIPC cohort, for being protected from AKI, was 46.2 ng/mL (sensitivity 0.69, specificity 0.76). It has been shown that HMGB1 at concentrations of 0.5 μg/mL might enhance neutrophil ROS production in the lung following hemorrhagic shock (35). We analyzed TNF-α release and did not find any significant effect after stimulation with HMGB1 at a concentration of 0.1 μg/mL, which we observe to induce transient, protective cell cycle arrest in renal tubular epithelial cells (Supplemental Figure 3A). Furthermore, the preconditioning of isolated neutrophils with HMGB1 (0.1 μg/mL) did not significantly modulate TNF-α–induced ROS production (Supplemental Figure 3B).

### HMGB1 induces transient TLR4-dependent cell cycle arrest in renal tubular epithelial cells.

**Figure 5:**
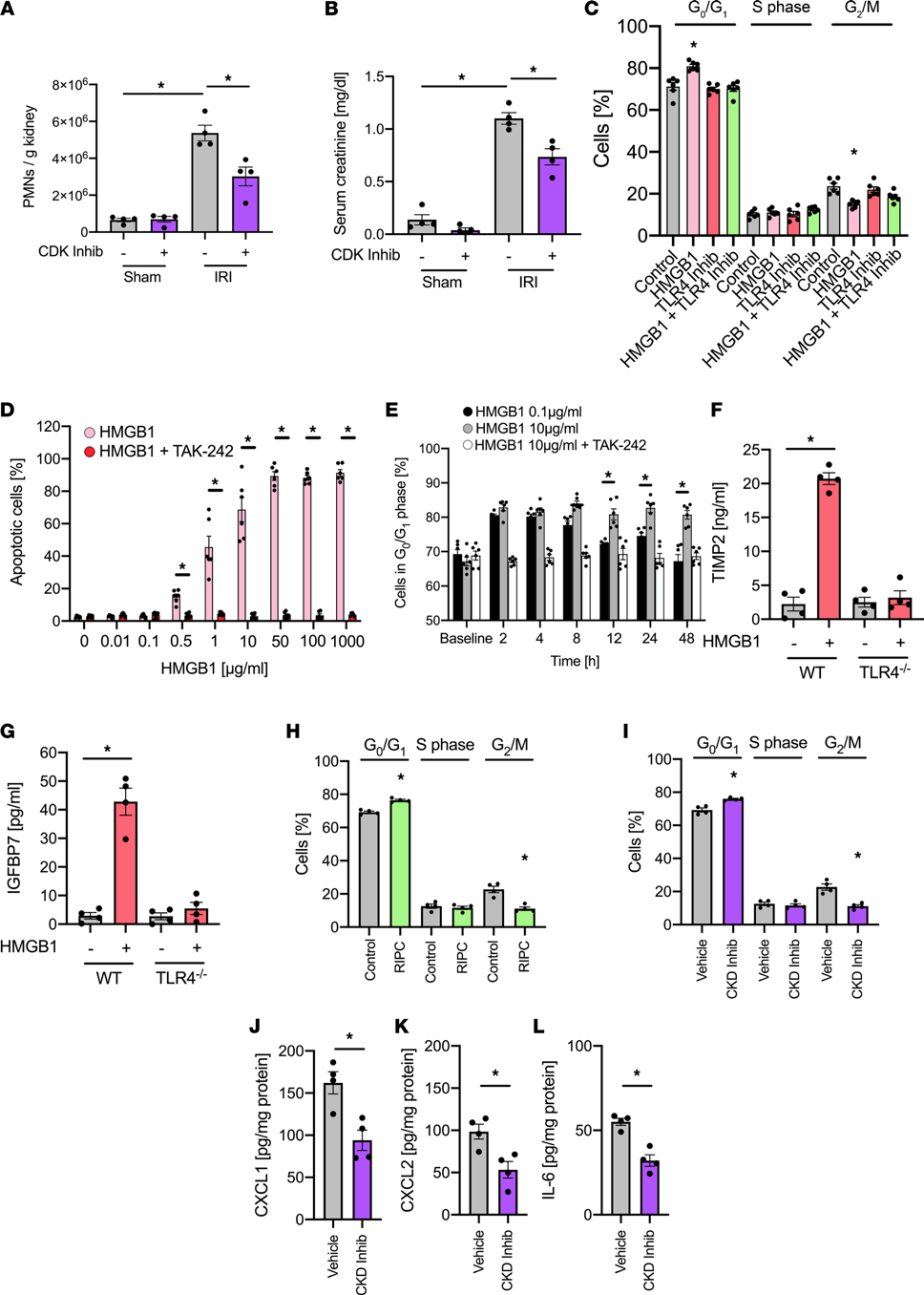
HMGB1 induces TLR4-dependent cell cycle arrest in renal tubular epithelial cells. After induction of general anesthesia, renal IRI was induced in WT mice by clamping of the renal pedicles for 32 minutes. Some mice received a cyclin-dependent kinase (CDK) inhibitor (PD0332991, 100 mg/kg) or a control before inducing renal IRI. Twenty-four hours after the surgery, mice were sacrificed. (**A**) The recruitment of neutrophils (PMNs) into the kidney was analyzed by flow cytometry (*n* = 4). (**B**) Serum creatinine levels were measured by a photometric assay (*n* = 4). (**C**) Isolated murine renal tubular epithelial cells were treated with control, HMGB1 (0.1 μg/mL), a TLR4 inhibitor (TAK-242), or HMGB1 together with TLR4 inhibitor in vitro for 24 hours. Cell cycle analysis was performed by measuring cellular DNA content by flow cytometry (*n* = 6). (**D**) Isolated murine renal tubular epithelial cells were treated with different concentrations of HMGB1 in presence or absence of of TLR4 inhibitor (TAK-242), and cell apoptosis was analyzed by propidium iodide/annexin V staining and flow cytometry (*n* = 6). (**E**) Isolated murine renal tubular epithelial cells were treated with HMBG1 0.1 μg/mL, HMGB1 10 μg/mL, or HMGB1 10 μg/mL plus TLR4 inhibitor (TAK-242) for 1 hour. The proportion of cells in G_0_/G_1_ phase was analyzed by measuring cellular DNA content by flow cytometry (*n* = 6). (**F** and **G**) Isolated murine renal tubular epithelial cells from WT and TLR4^fl/fl^/Ksp-Cre^+/T^ mice were treated with HMGB1 and TIMP-2 (**F**) and IGFBP7 (**G**) released into the supernatant were analyzed by ELISA (*n* = 4). (**H** and **I**) Cell cycle arrest in renal tubular epithelial cells was analyzed by flow cytometry (*n* = 4). (**J**–**L**) The levels of the chemokines CXCL1 (**J**), CXCL2 (**K**), and IL-6 (**L**) in kidney tissue homogenisates were analyzed by ELISAs (*n* = 4). Mann-Whitney *U* test (**J**–**L**) and 1-way ANOVA followed by Bonferroni testing (**A**–**I**) were used for statistical analysis; *P < 0.05.

In order to investigate whether the induction of cell cycle arrest before renal IRI also has a renoprotective effect in vivo, we injected a potent CDK4/6 inhibitor (PD 0332991, 100 mg/kg) to interrupt the cyclin kinase–dependent cell cycle regulation and induction of G_1_ cell cycle arrest in WT mice before inducing renal IRI (36), which attenuated AKI severity, as shown by a reduction in renal neutrophil recruitment and by serum creatinine levels in the plasma (Figure 5, A and B). In order to test whether low concentrations of HMGB1 induce cell cycle arrest, we used an in vitro cell culture system with isolated murine renal tubular epithelial cells and performed flow cytometry–based cell cycle analysis following the stimulation with HMGB1 (5 ng/mL). To characterize the proximal tubular cells, we analyzed the mRNA expression of prominin-1 (marker for proximal tubules), aquaporin-2 (marker of collecting ducts), and CD31 (marker of endothelial cells). We confirmed by quantitative PCR (qPCR) that the isolated cells were expressing prominin-1 but not Aqp2 or CD31 (Supplemental Figure 4A), thus demonstrating the specificity of the isolation procedure (37). The results from these experiments show that HMGB1 induces cell cycle arrest, with more cells in the cell population persisting in the G_0_/G_1_ phase and less cells proceeding to the G_2_/M phase (Figure 5C). Interestingly, the effect of HMGB1 was abolished by cotreatment with a TLR4 inhibitor (TAK-242, 1 μM), indicating that HMGB1 binding to TLR4 on renal cells is necessary to induce cell cycle arrest in the G_0_/G_1_ phase (Figure 5C) (38). Importantly, blocking RAGE or CXCR4, which are also receptors for HMGB1, did not affect the induction of G_1_ cell cycle arrest by HMGB1 (Supplemental Figure 4B). The TLR4 inhibitor alone did not have an effect on the induction of cell cycle arrest. The HMGB1 concentration we used is lower compared with HMGB1 concentrations observed under conditions of systemic inflammation and did not provoke significant induction of apoptosis in isolated renal tubular epithelial cells (Figure 5D). In contrast, concentrations of HMGB1 higher than 0.5 μg/mL did induce apoptosis, which was blocked by coincubation with the TLR4 inhibitor TAK-242 (Figure 5D). In a time-course analysis, the incubation of isolated renal tubular epithelial cells with HMGB1 (0.1 μg/mL) induced a transient cell cycle arrest with an increased percentage of cells in the G_1_ phase over a period of 8 hours. This transient cell cycle arrest eventually resolved after 8 hours, and the percentage of cells arrested in the G_1_ cell cycle phase decreased again (Figure 5E). In contrast, cells that were exposed to a higher HMGB1 concentration of 10 μg/mL for 1 hour, a concentration that we also found to induce cell apoptosis (Figure 5D), showed persistent cell cycle arrest of the observation period of 48 hours (Figure 5E). The coincubation of HMGB1 (0.1 μg/mL) together with TAK-242 did not induce a transient cell cycle arrest, suggesting that HMGB1 binding to TLR4 on renal tubular epithelial cells is required for this process. We further observed that isolated WT murine renal tubular epithelial cells stimulated with HMGB1 in vitro showed elevated levels of TIMP-2 and IGFBP7 in the supernatant compared with control, which were absent in cells isolated from TLR4-deficient animals (Figure 5, F and G). Accordingly, the application of RIPC caused a significant increase in cells undergoing a transient G_0_/G_1_ cell cycle arrest and less cells in the G_2_/M phase of renal tubular epithelial cells (TECs) isolated from WT mice 4 hours after RIPC procedure compared with renal TECs from control mice not subjected to RIPC (Figure 5H). This effect lasted for up to 8 hours and was not detectable after 12 or 24 hours (data not shown). In addition, application of a CDK inhibitor (PD 0332991, 100 mg/kg) in vivo before the induction of IRI led to a cell cycle arrest with an increased percentage of cells remaining in the G_0_/G_1_ phase (Figure 5I), and it reduced the release of the proinflammatory mediators CXCL1, CXCL2, and IL-6 (Figure 5, J–L). In order to analyze possible effects of CDK inhibition on the cell cycle state of circulating neutrophils and BM-resident CD45^+^ leukocytes, we isolated the cells after administration of a CDK inhibitor and analyzed the cell cycle state. No significant alterations between the groups was observed (data not shown).

### HMGB1 induces NF-κB and AMPKα activation.

**Figure 6:**
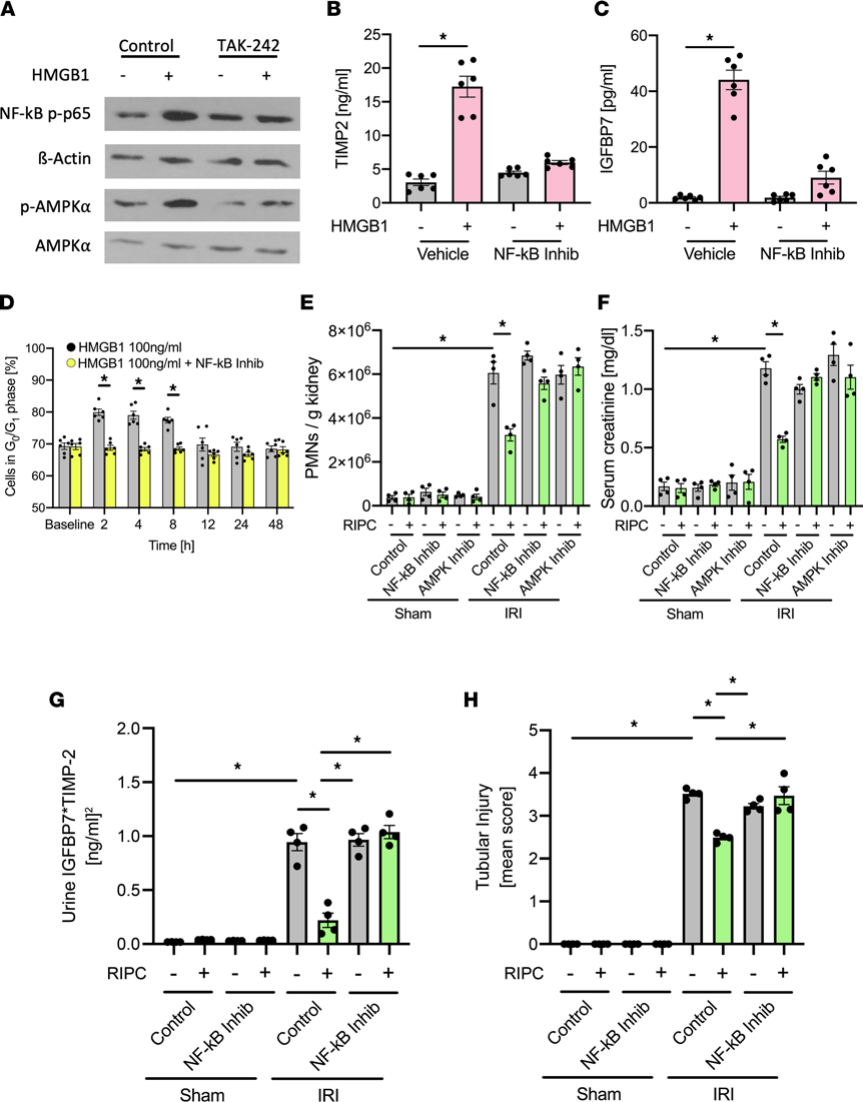
HMGB1 induces NF-κB and AMPKα activation. (**A**) Isolated murine renal tubular epithelial cells were incubated with HMBG1 (0.1 μg/mL) or HMGB1 plus TLR4 inhibitor (TAK-242). Activation of NF-κB and AMPKα was detected by Western blotting for NF-κB p-p65 and β-actin as loading control, as well as p-AMPKα and total AMPKα (exemplary blots). Isolated murine renal tubular epithelial cells were incubated with HMBG1 (0.1 μg/mL) or HMGB1 plus NF-κB inhibitor (Bay-117082). (**B** and **C**) TIMP-2 and IGFBP7 were analyzed by ELISAs (*n* = 6). (**D**) Isolated murine renal tubular epithelial cells were treated with HMBG1 (0.1 μg/mL) or HMGB1 in combination with NF-κB inhibitor (Bay-117082). The proportion of cells in G_0_/G_1_ phase was analyzed by measuring cellular DNA content by flow cytometry (*n* = 6). After induction of general anesthesia WT mice received either 3 cycles RIPC or control procedure. Some mice received a NF-κB inhibitor (Bay-117082, 10 mg/kg i.p.) or AMPKα inhibitor before RIPC. Twenty-four hours after IRI induction, mice were sacrificed. (**E**) The recruitment of neutrophils (PMNs) into the kidney was analyzed by flow cytometry (*n* = 4). (**F**) Serum creatinine levels were measured by a photometric assay (*n* = 4). (**G**) The biomarkers TIMP-2 and IGFBP7 were measured in urine samples 24 hours after inducing renal IRI. (**H**) Renal tubular injury score was assessed based on histology (*n* = 4). One-way ANOVA followed by Bonferroni testing was used for statistical analysis; *P < 0.05.

HMGB1 activates NF-κB, and NF-κB activation may also induced cell cycle arrest (39). To test if NF-κB signaling might be involved in the regulation of HMGB1-mediated renal protection from AKI, we stimulated isolated renal tubular epithelial cells with HMGB1 (0.1 μg/mL) and observed increased phosphorylation of NF-κB p-p65 (Figure 6A), which is a prerequisite for NF-κB release, nuclear translocation, and NF-κB–induced gene transcription. Interestingly, coincubation with the TLR4 inhibitor TAK-242 nearly completely abrogated NF-κB p-p65 activation (Figure 6A). AMPKα is involved in NF-κB activation, but it is unknown if it plays a role following HMGB1 stimulation. We found AMPKα to be phosphorylated in isolated renal tubular epithelial cells after treatment with HMGB1 (0.1 μg/mL), which again was reversed by coincubation with the TLR4 inhibitor TAK-242 (Figure 6A). HMGB1 stimulation also led to a release of TIMP-2 and IGFBP7, which are both involved in G_1_ cell cycle arrest (Figure 6, B and C). Blocking NF-κB activation inhibited the transient (protective) induction of G_1_ cell cycle arrest in renal tubular epithelial cells needed for protection from AKI (Figure 6D). When administered to mice in vivo prior to RIPC application, NF-κB inhibition and AMPKα inhibition reversed the protective effect of RIPC and caused increased neutrophil recruitment into the kidney (Figure 6E) and elevated serum creatinine levels (Figure 6F). Furthermore, NF-κB inhibition increased urinary concentrations of TIMP-2 and IGFBP7 (Figure 6G and Supplemental Figure 7) and increased renal tubular injury (Figure 6H) 24 hours after the induction of AKI.

### Sema5b upregulation is involved in HMBG1-mediated renal protection.

**Figure 7:**
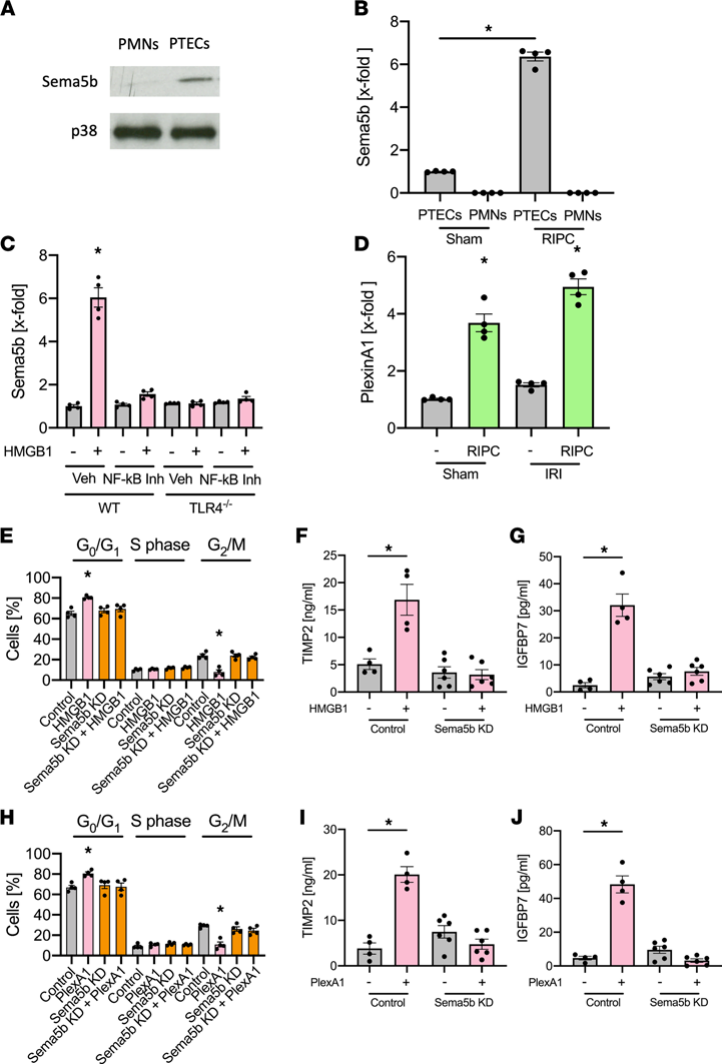
Sema5b upregulation is involved in HMBG1-medieated renal protection. (**A**) Isolated murine renal tubular epithelial cells were lysed, and Sema5b and p38 (as a loading control) was detected by Western blotting (exemplary blot from *n* = 4 independent experiments). (**B**) Murine renal tubular epithelial cells and neutrophils were isolated from WT mice 4 hours after sham or RIPC application, and Sema5b expression was analyzed by qPCR (*n* = 4). (**C**) Murine renal tubular epithelial cells were isolated from WT or TLR4/Ksp-Cre mice 4 hours after injection of rHMGB1 and/or a NF-κB inhibitor (Bay-117082, 10 mg/kg i.p.), and Sema5b expression was analyzed by qPCR (*n* = 4). (**D**) Isolated murine renal tubular epithelial cells were isolated from WT mice 4 hours after RIPC procedure, and Sema5b expression was detected by qPCR (*n* = 4). (**E**) Isolated WT or Sema5B-KD murine renal tubular epithelial cells were treated with control or HMGB1 (0.1 μg/mL) in vitro for 24 hours. (**F** and **G**) Cell cycle analysis was performed by measuring cellular DNA content by flow cytometry, and TIMP-2 (**F**) and IGFBP7 (**G**) released into the supernatant were analyzed by ELISA (*n* = 4–6). (**H**) Isolated WT or Sema5b-KD murine renal tubular epithelial cells were treated with control or PlexinA1 in vitro for 24 hours. (**I** and **J**) Cell cycle analysis was performed by measuring cellular DNA content by flow cytometry and TIMP-2 (**I**) and IGFBP7 (**J**) released into the supernatant were analyzed by ELISA (*n* = 4–6). One-way ANOVA followed by Bonferroni testing was used for statistical analysis; *P < 0.05.

Semaphorins are in the regulation of various cellular functions (40). We observed a 5.2-fold increase in Semaphorin 5b (Sema5b) expression after RIPC application in renal tubular epithelial cells (Figure 2, C and D) and could confirm Sema5b to be expressed on isolated PTECs — but not neutrophils (Figure 7, A and B). Interestingly, Sema5b expression on PTECs was significantly increased following RIPC and after HMGB1 injection, but increased Sema5b expression was lacking following NF-κB inhibition or in TLR4/Ksp-Cre mice (Figure 7, B and C), indicating that RIPC-elicited increased HMBG1 levels mediate Sema5b upregulation on PTECs in a TLR4-dependent manner. Sema5b action involves binding to its endogenous ligand PlexinA1 (41). Interestingly, while Sema5b was approximately 5-fold upregulated in TECs following RIPC (highlighted in Figure 2D), we did not find differential regulation of PlexinA1 mRNA by RNA-Seq analysis in TECs (*P* = 0.059). However, we performed a selective qPCR analysis and could observe significant upregulation of PlexinA1 in TECs after RIPC procedure; this upregulation was even more pronounced after IRI induction (Figure 7D). Furthermore, HMGB1 administration was able to induced cell cycle arrest, with more cells in the cell population persisting in the G_0_/G_1_ phase and less cells proceeding to the G_2_/M phase in isolated WT PTECs, whereas the effect of HMGB1 was abolished in Sema5b-KD PTECs, indicating that Sema5b on renal cells is necessary to induce protective cell cycle arrest in the G_0_/G_1_ phase (Figure 7E). Furthermore, isolated WT PTECs stimulated with HMGB1 in vitro showed elevated levels of TIMP-2 and IGFBP7 in the supernatant compared with control, and this elevation was absent in Sema5b-KD cells (Figure 7, F and G). Similarly, the incubation with recombinant PlexinA1 induced protective cell cycle arrest and increases TIMP-2 and IGFBP7 levels in the cell supernatant, and these effects were abolished in Sema5b-KD cells (Figure 7, H–J).

### Sema5b regulates RIPC-induced renal protection from IRI in vivo.

**Figure 8:**
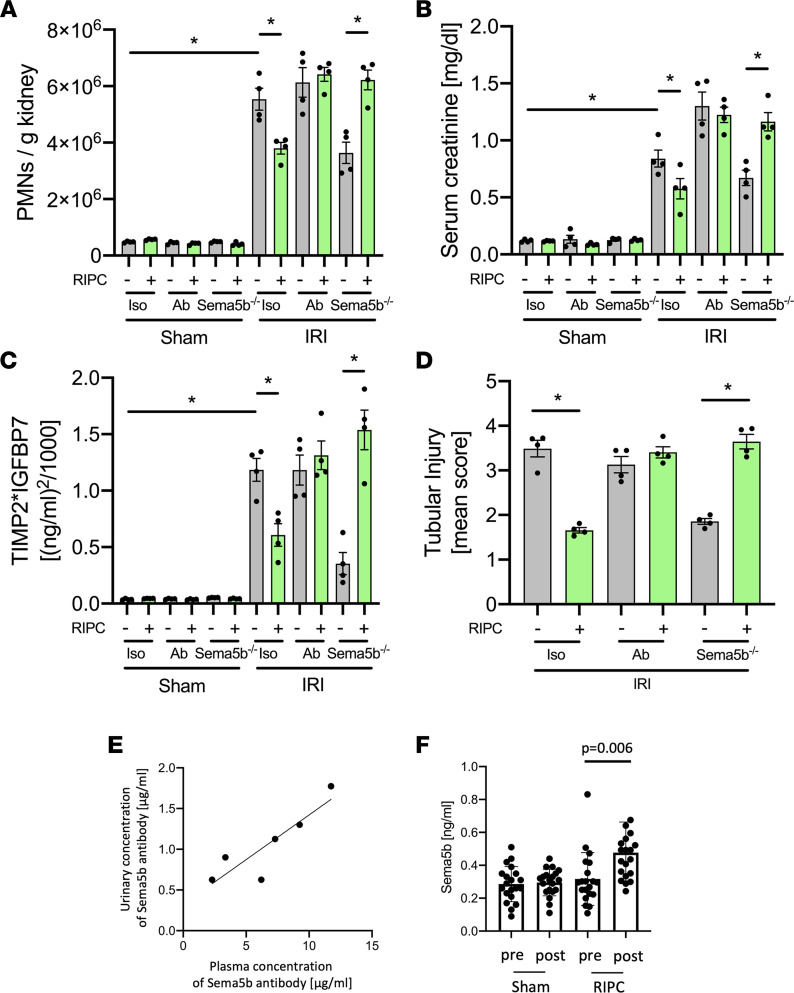
Sema5b regulates RIPC-induced renal protection from IRI in vivo. After induction of general anesthesia, renal IRI was induced in WT and Sema5b-deficient mice by clamping of the renal pedicles for 32 minutes. Some mice received a blocking Sema5b antibody (50 μg/mouse) or were subjected to RIPCs before IRI induction. Twenty-four hours after the surgery, mice were sacrificed. (**A**) The recruitment of neutrophils (PMNs) into the kidney was analyzed by flow cytometry (*n* = 4). (**B**) Serum creatinine levels were measured by a photometric assay (*n* = 4). (**C**) The biomarkers TIMP-2 and IGFBP7 were measured in urine samples 24 hours after renal IRI (*n* = 4). (**D**) Quantification of histological tubular injury (*n* = 4). (**E**) Detection of injected Sema5b antibody in the plasma and urine by sandwich ELISA (*n* = 6). (**F**) Urinary levels of Sema5b in cardiac surgery patients from the RIPCrenal trial before and 4 hours after RIPC application (*n* = 20/group). One-way ANOVA followed by Bonferroni testing was used for statistical analysis; *P < 0.05.

Both the pharmacological blockade of and genetic ablation of Sema5b abolished the protective effect of RIPC and led to increased neutrophil recruitment into the kidney, elevated serum creatinine levels, and persistently high TIMP-2 and IGFBP7 urine levels 24 hours after IRI induction, indicative of increased renal injury (Figure 8, A–D, and Supplemental Figure 7). In this in vivo experiment, the blocking Sema5b antibody was detectable in proportional levels in the urine of the mice associated with the plasma levels after systemic injection (Figure 8E).

In order to confirm that Sema5b is involved in RIPC-elicited amelioration of AKI, we analyzed the concentrations of Sema5b before and 4 hours after RIPC or sham application in a cohort of cardiac surgery patients of the RIPCrenal trial (31). Urinary Sema5b concentrations were significantly higher in patients receiving RIPC compared with sham patients (Figure 8F).

### TIMP-2 is required for HMGB1- and RIPC-mediated renal protection.

**Figure 9:**
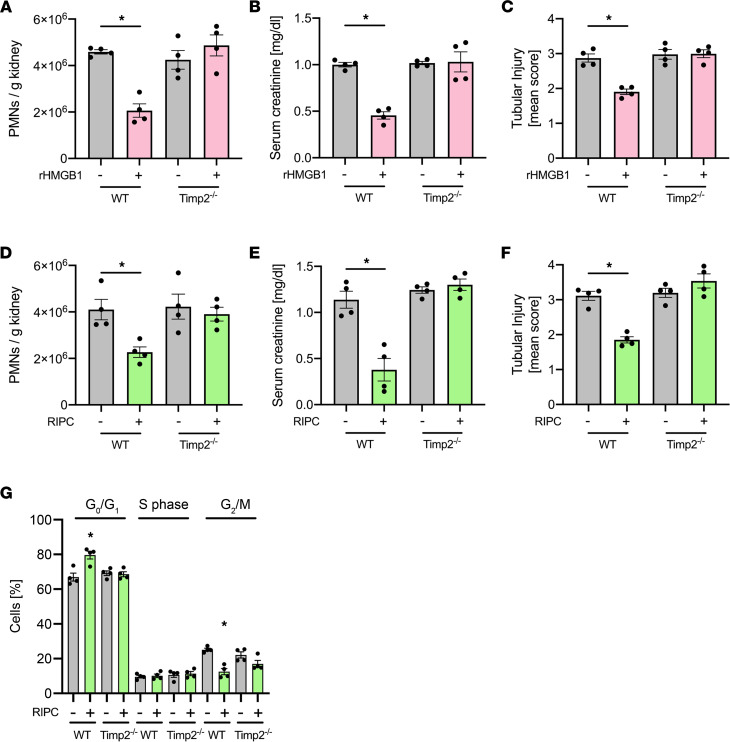
TIMP-2 is required for HMGB1- and RIPC-mediated renal protection. After induction of general anesthesia, WT control and TIMP-2^–/–^ mice were pretreated with rHMBG1 or control, and renal IRI was induced by clamping of the renal pedicles for 32 minutes. Twenty-four hours after the surgery, mice were sacrificed. (**A**) The recruitment of neutrophils (PMNs) into the kidney was analyzed by flow cytometry (*n* = 4). (**B**) Serum creatinine levels were measured by a photometric assay (*n* = 4). (**C**) Renal tubular injury score was assessed based on histology (*n* = 4). In an analog set of experiments, WT control and TIMP-2^–/–^ mice received RIPC or sham procedure, and renal IRI was induced by clamping of the renal pedicles for 32 minutes. Twenty-four hours after the surgery, mice were sacrificed. (**D**) The recruitment of neutrophils (PMNs) into the kidney was analyzed by flow cytometry (*n* = 4). (**E**) Serum creatinine levels were measured by a photometric assay (*n* = 4). (**F**) Renal tubular injury score was assessed based on histology (*n* = 4). (**G**) Cell cycle arrest in renal tubular epithelial cells was analyzed by flow cytometry (*n* = 4). One-way ANOVA followed by Bonferroni testing was used for statistical analysis; *P < 0.05.

In an attempt to investigate if TIMP-2 not only serves as a biomarker for AKI, but may itself also be involved in the protection from AKI, we pretreated WT and TIMP-2–deficient mice with HMGB1 and subsequently induced AKI using IRI in these animals. In contrast to WT mice, the administration of HMGB1 had no effect in TIMP-2–deficient mice (Figure 9, A–C). Furthermore, RIPC was also unable to lead to any significant effect on neutrophil recruitment, serum creatinine levels, or histology (Figure 9, D–F) in TIMP-2–deficient mice. In addition, the genetic deletion of TIMP abrogated the RIPC-induced G_0_/G_1_ cell cycle arrest (Figure 9G). These findings indicate that TIMP-2 is required for the conveyance of renal protection from IRI-induced AKI elicited by HMGB1 and RIPC.

### RIPC also ameliorates glycerol-induced AKI.

The application of RIPC 15 minutes before the induction of glycerol-induced AKI significantly decreased neutrophil recruitment into the kidney, serum creatinine and urinary renal injury biomarker levels, and histological evidence for renal tubular injury (Supplemental Figure 5, A and B). These data indicate that RIPC may also mediate renal protection from glycerol-induced AKI. The transcription factor p53 induces the upregulation of the cyclin kinase inhibitor p21, which is known to mediate apoptosis in some AKI models, including glycerol-induced and IRI-induced AKI (42). We performed qPCR to investigate p53/p21 activation in the kidney and found both elements to be significantly upregulated after RIPC procedure compared with untreated controls (Supplemental Figure 6).

## Discussion

Our study demonstrates that the release of the endogenous alarmin HMGB1 following RIPC causes the induction of a transient cell cycle arrest in renal tubular epithelial cells by binding of HMGB1 to its receptor TLR4. On a molecular level, this induces the activation of NF-κB by phosphorylation the p65 subunit and the upregulation of Sema5b on PTECs by inducing a transient, protective cell cycle arrest.

RIPC therapy has been controversial. We demonstrated in a multicenter trial that RIPC in high-risk patients undergoing cardiac surgery reduces the occurrence of AKI (24). However, other trials have not shown positive effects, possibly related to the inclusion of lower-risk patients and/or the use of interfering drugs such as propofol (29, 30). Thus, a better understanding of the mechanisms of RIPC-mediated renal protection is essential. As in our clinical study, the application of RIPC in mice induced an early increase of HMGB1 and an early and transient increase of the G_1_ cell cycle arrest markers TIMP-2 and IGFBP7 (24). Interestingly, not all patients receiving the RIPC procedure responded with an early increase of TIMP-2 and IGFBP7 levels before the surgical insult. However, the group of RIPC patients that did respond with early increased levels of TIMP-2 and IGFBP7 after application of RIPC had significantly decreased rates of AKI after the surgery (24). Thus, it is striking that RIPC did not effectively induce a transient, protective cell cycle arrest (as indicated by transient, early increase of TIMP-2 and IGFBP7) in all patients, but if a patient responded to RIPC treatment, the procedure led to protection of the kidneys from subsequent injury. In contrast to the early increase of the cell cycle arrest biomarkers immediately after RIPC application, patients who later developed AKI after the surgical procedure showed a prolonged increase of TIMP-2 and IGFBP7, which may be linked to persistent cell cycle arrest in the kidney and be predictive for the development of an AKI. These findings fit very well with our observations in the murine system where we showed that RIPC caused an elevation of HMGB1 levels in the serum and that pretreatment with HMGB1 alone was sufficient to induce a protective, transient cell cycle arrest and provide renal protection similar to RIPC.

HMGB1 is involved in the pathogenesis of numerous inflammatory processes (43). During systemic inflammation, HMGB1 is also a mediator involved in excessive immune system activation and collateral organ tissue damage (44). Thus, this raises the question as to how HMGB1 released in response to RIPC would elicit renal protection from subsequent renal IRI. Strikingly, HMGB1 is released after RIPC only in very low concentrations compared with the relatively high concentrations of HMGB1 during systemic inflammation (45). These low concentrations appear to be sufficient to induce TLR4-dependent low-grade NF-κB signaling and a transient cell cycle arrest in renal tubular epithelial cells. This is also in line with our observation that RIPC alone induced a low-level increase of HMGB1 in the serum, inducing a protective, transient cell cycle arrest, whereas the IRI causes higher concentrations of HMGB1 in the serum at later time points (>12 hours), and these higher HMGB1 concentrations induced persistent, deleterious cell cycle arrest. The administration of higher doses of rHMGB1 also induced kidney injury. Of note, the unique environment in the renal tubules where concentrated solute, including filtered HMGB1, are available to cell receptors may help explain how very low plasma concentrations of HMGB1 are still pharmacologically active in the kidney. Furthermore, the moderately increased HMGB1 plasma levels after RIPC return to baseline values as soon as 1–4 hours after RIPC. Thus, this tight time course may mitigate the potentially negative effects of prolonged HMGB1 signaling. HMGB1 may also promote the regeneration of nerve cells, remodeling of blood vessels, and recovery of neurological function in the late infarct stage (46). Thus, the molecular mechanisms of HMGB1 may well go beyond inflammation, and further studies should address this. TLR4 on different cell types has different functions in the pathophysiology of IRI-induced AKI (47). Our data suggest that TLR4 on PTECs is not implicated in the development of AKI, since depleting this receptor did not change the severity of AKI. However, TLR4 expression on PTECs is important for mediating the renoprotective effect of RIPC. In contrast, TLR4 on neutrophils is critically involved in the development of AKI because blocking this receptor on PMNs resulted in the attenuation of AKI.

The pathophysiology of AKI involves cell cycle arrest (10). The urinary levels the cell cycle arrest markers TIMP-2 and IGFBP7 have been demonstrated to be valuable biomarkers for AKI (48). Transient G_1_ cell cycle arrest indicated by early and reversible increase of TIMP-2 and IGFBP7 after RIPC application are associated with postoperative renal protection, whereas late and persistent cell cycle arrest is associated with a higher incidence of AKI and renal injury (24). On a cellular level, transient G_1_ cell cycle arrest appears to represent a self-defense program that enables renal epithelial cells to withstand limited periods of cellular stress— e.g., due to diminished oxygen and energy metabolite supply (49). Here, we demonstrate that HMGB1 binding to TLR4 on renal tubular epithelial cells induces G_1_ cell cycle arrest through activation of NF-κB and AMPKα, and subsequent NF-κB activation has been shown to be implicated in the several inflammatory processes (50). Furthermore, NF-κB activation may mediate G_1_ cell cycle arrest, which is also in accordance with our data (39). Semaphorins are currently recognized to control a wide range of cellular functions in various tissues, including the immune system and in the kidney (51, 52). Since Sema5b was indicated by our RNA-Seq analysis as one of the most upregulated genes in PTECs following RIPC, we found Sema5b and the interaction with its endogenous ligand PlexinA1 to be involved in the RIPC- and HMBG1-elicited induction of transient cell cycle arrest and renal protection. This was also supported by the finding that Sema5b levels were increased after application of RIPC in cardiac surgery patients. Our data are supported by previous reports on Sema5b expression in the human kidney (53). However, to our knowledge, this is the first report to show the renal protective effect of upregulated Sema5b expression in PTECs; thus, the underlying molecular mechanism of the Sema5b-PlexinA1 interaction warrants further research.

Interestingly, our study also shows that the genetic deletion of TIMP-2 abolished the protective effect of RIPC and HMGB1 pretreatment. Thus, TIMP-2 appears to not only serve as a cell cycle arrest biomarker, but it also seems to be actively involved in mediating transient, protective cell cycle arrest after RIPC application or HMGB1 pretreatment. This finding is also supported by previous studies reporting cell cycle regulation by TIMP-2 (54). To this end, however, it remains unclear how TIMP-2 is mechanistically involved in the pathogenesis of AKI and/or the molecular mechanism providing renal protection from AKI after application of RIPC; further research on this topic is needed.

RIPC possesses the advantage that the intervention is harmless and cheap. Which predisposing factors determine if a patient benefits from RIPC or whether alternative RIPC protocols (e.g., intensity, duration) will result in greater or less response rates is not well known to date and has to be investigated in further studies (31). Here, we unveil that HMGB1 release leading to TLR4-dependent AMPKα and NF-κB activation, followed by TIMP-2–dependent cell cycle inhibition, is part of the underlying molecular signaling pathways governing the conveyance of renal protection by RIPC. This knowledge is important to develop further therapeutic approaches and may also lead to an increase in the proportion of patients who benefit from this preconditioning procedure.

## Methods

### Animals and reagents.

We used 8- to 12-week-old male C57BL/6, Ksp-Cre^+/T^, TLR4^fl/fl^,TIMP-2^–/–^, and Sema5b^–/–^ mice and littermate controls (55–57). The mice were kept in a barrier facility under specific pathogen–free (SPF) conditions. All animal experiments were approved by the institutional review board (Landesamt für Natur-, Umwelt- und Verbraucherschutz Nordrhein-Westfalen; LANUV NRW). Recombinant, full-length disulfide HMGB1 (catalog HM-121) and BoxA (catalog HM-012, produced in *E. coli*, tested LPS free) were obtained from HMGBiotech. The NF-κB inhibitor Bay-117082 was purchased from Merck. Unless otherwise stated, all other reagents were obtained from Sigma-Aldrich.

### RIPC.

RIPC was applied by placing a customized pressure cuff (Kent Scientific) around the hind limb of an anesthetized mouse. The cuff was inflated to 200 mmHg (well above arterial systolic pressure in anesthetized mice) 3 times for 5 minutes with a 5-minute break between the treatments. RIPC treatment was performed 15 minutes prior to induction of AKI.

### Murine AKI model by renal IRI and glycerol injection.

The IRI model has been described previously (58). Briefly, mice were anesthetized with i.p. injections of ketamine (125 mg/kg) and xylazine (12.5 mg/kg) and placed on a heating pad to maintain body temperature. In animals undergoing renal ischemia-reperfusion, kidneys were surgically exposed through access by the retroperitoneal route, and both renal pedicles were clamped off for 32 minutes with hemostatic microclips. After clamp removal, kidneys were checked for a change in color within 3 minutes to ensure reperfusion. In animals subjected to sham operation, the surgical procedure was identical except that no clamps were applied. After surgery, animals were kept under a heating lamp to maintain body temperature and had free access to food and water. Ischemia was induced by clamping of the renal pedicles about 15 minutes after completion of the RIPC procedure. Some mice received rHMGB1 (0.3, 3, or 30 μg/mouse i.v.) before the induction of renal IRI. Glycerol-induced AKI was performed as previously described (42). Some mice were neutrophil depleted by using a depleting antibody (clone RB6-8C5, produced in-house, 30 mg/mouse i.p. 24 hours before the experiment).

### Quantification of renal neutrophil recruitment, serum creatinine levels, and biomarkers.

After 24 hours, the mice were euthanized and blood samples were obtained by heart puncture. After cutting of the vena cava, mice were perfused through the left ventricle, and flushing of kidneys was judged by color change. Kidneys were harvested to determine the number of neutrophils in the kidney. Serum creatinine levels were determined by using a creatinine assay (Diazyme) according to the manufacturer’s protocol. Neutrophil recruitment into the kidneys was determined by flow cytometry as previously described (58). Briefly, after flushing and removal from the body, the kidneys were mechanically minced and enzymatically digested by incubation with collagenase, hyaluronidase, and DNase. After 1 hour, the homogenized kidney tissue was passed over a cell strainer (mesh size 70 μm). Staining was performed with fluorescently labeled antibodies against Ly6G (clone 1A8, BD Biosciences), Ly6B:2 (clone 7/4, Bio-Rad), and CD45 (clone 30-F11, BD Biosciences), and samples were run on a flow cytometer (BD FACSCanto II). Neutrophils were identified as CD45^+^Ly6G^+^Ly6B:2^+^ cells. Absolute neutrophil counts in the samples were analyzed by the use of FACS counting beads. HMGB1, TIMP-2, and IGFBP7 were measured by ELISA kits according to the manufacturers instruction (R&D Systems).

### Measuring chemokine and protein concentrations.

Chemokine concentrations in homogenized tissue samples, supernatants, or serum were measured by ELISAs according to the manufacturer’s protocol (R&D Systems). Sema5b concentrations in human urine were analyzed by ELISAs according to the manufacturer’s protocol (BIOZOL).

### Primary culture of mouse proximal tubular epithelial cells.

Renal tubular epithelial cells were isolated as previously described, with some modifications (37). Briefly, kidneys were removed from mice after sacrifice by cervical dislocation; the capsule was removed, and the organ was mechanically minced. The tissue was transferred to DMEM-F12 containing 1 mg/mL collagenase type II. Kidneys were digested by incubation in collagenase at 37°C for 10 minutes; they were then vortexed for 1 minute and incubated for a further 10 minutes, followed by another vortexing round for 1 minute. The cell solution was transferred through a 30 μm nylon mesh filter, which was rinsed with medium. The solution was collected and centrifuged at 500*g* for 10 minutes at room temperature. The cells were incubated with prominin-1 antibody–coated (clone 13A4, Invitrogen) magnetic microbeads and isolated by use of magnetic separation in an LS column (Miltenyi Biotec). The isolated cells were resuspended in medium on tissue culture plates and incubated at 37°C with 5% CO_2_. The fibroblast content in the tubular epithelial cell culture after the isolation procedure was < 2%.

### Histological examination.

Kidneys were fixed in 4% formaldehyde, embedded in paraffin, and sectioned at 2 μm for H&E staining. Histopathological examination was performed by pathologists blinded to the conditions. Tubular injury was scored by estimating the percentage of tubules in the cortex or the outer medulla that showed epithelial necrosis or had luminal necrotic debris and tubular dilatation as follows: 0 = none; 1 ≤ 5%; 2 = 5%–30%; 3 = 30%–75%; and 4 ≥ 75% (59). For each slide, at least 10 fields were reviewed at a magnification of 200×.

### Western blot.

Isolated murine renal tubular epithelial cells (see description of isolation procedure above) were lysed with RIPA buffer (60). Lysates were boiled with Laemmli sample buffer, run on 10% SDS-PAGE gels and immunoblotted using antibodies against NF-κB p-p65 (clone 93H1, Cell Signaling Technology), pAMPKα (clone D4D6D, Cell Signaling Technology), AMPKα (clone D63G4, Cell Signaling Technology), and p38 (catalog 9212, Cell Signaling Technology); Semaphorin 5b (catalog NBP2-56604, Novus Biologicals); or β-actin (clone AC-15, Sigma-Aldrich). Immunoblots were developed using an ECL system (GE Healthcare).

### Analysis of cell cycle arrest and apoptosis.

Cell cycle analysis was performed by first fixing the cells using cold 70% ethanol for 30 minutes and washing them twice in PBS with centrifugation at 500*g* for 10 minutes at room temperature. RNA was degraded by RNase A (0.1 mg/mL, Sigma-Aldrich) treatment, and nuclear DNA was stained by propidium iodide (1 μg/mL, Invitrogen). Cell cycle analysis was performed using a flow cytometer (BD FACSCanto II), and doublets were excluded by gating on single cells. Doublets were excluded by FACS FSC-A/FSC-H gating. Apoptosis was analyzed by staining for Propidium iodide and annexin V, followed by flow cytometric analysis.

### Effectiveness of RIPC in human cardiac surgery patients.

We included human data from the previous RenalRIP trial, and CONSORT-compliant reporting of the study design of this trial has been published before (24). Briefly, this was a multicenter, randomized-controlled trial including 240 patients undergoing cardiac surgery at high risk for AKI (ID DRKS00005333). Patients were randomly allocated to receive either RIPC (3 cycles of 5-minute cuff inflation to 200 mmHg or ≥ 50 mmHg above the systolic pressure followed by 5-minute cuff deflation) or sham-RIPC. [TIMP-2]*[IGFBP7] and HMGB1 levels were measured immediately after the RIPC/sham-RIPC intervention prior to cardiopulmonary bypass. The primary outcome was occurrence of AKI within 72 hours after cardiac surgery.

### ROS production.

ROS production was analyzed as described previously (61). Briefly, isolated neutrophils were plated on poly-RGD–coated (20 mg/mL) 96-well plates with CaCl_2_ (1 mM), MgCl_2_ (1 mM), and cytochrome c (0.1 mM) in the presence of TNF-α (0.5 mg/mL) and/or SOD (superoxidedismutase, ~45 U), and plates were analyzed on a plate reader.

### RNA-Seq.

RNA was extracted from the sorted cells using a RNeasy Mini Kit (Qiagen). Total RNA samples were mixed with oligo-dT and deoxynucleotide triphosphates (dNTPs), incubated at 72°C, and immediately put back on ice. Reverse transcription into cDNA was performed based on polyA tail. The template was switched at the 5′ end of the RNA, and the full-length cDNA was amplified by PCR. The average molecule length was analyzed using the Agilent Technologies 2100 bioanalyzer instrument. For library construction, PCR products were purified and selected with the Agencourt AMPure XP-Medium kit. DNA was quantified by the Agilent Technologies 2100 bioanalyzer. The double-stranded PCR products were heat denatured and circularized by the splint oligo sequence. The single-strand circle DNA (ssCirDNA) were formatted as the final library. The library was amplified to make DNA nanoball (DNB), which had more than 300 copies of 1 molecule. The DNBs were loaded into the patterned nanoarray, and single-end 50-base reads were generated by sequencing by combinatorial Probe-Anchor Synthesis (cPAS). The RNA-Seq data underwent thorough quality control with FastQC and MultiQC (62), before sequencing adapter trimming was performed with the R package trim-galore. Read-mapping and quantification was performed with Salmon (63) using the Gencode (64) mouse reference transcriptome vM23. The Salmon quasiindex was created with a default k-mer length of 31 and the --gencode flag. The subsequent quantification was performed while accounting for sequence-specific biases like random hexamer priming, which often results in lower base-call quality of the first few bases of a read. Additional parameters included --validateMappings and --rangeFactorizationBins 4 to potentially improve the quantification accuracy. Additionally, the --gcBias flag was used to correct for the slightly skewed GC-content of the reads, which was observed in the quality control step. The transcript counts were summarized to gene level with tximport (65) and supplementary annotation data from Ensembl (66) through the biomaRt-package (67, 68). During this summarization step, allosomal and mitochondrial genes were excluded to decrease (sex-specific) biases. The differential expression analysis was carried out by DESeq2 (69) on the gene-level counts. The reported log fold changes were shrunk with apeglm (70) and *S* values were calculated for statistical significance filtering. For a significant differential gene, we required an *S* value below 0.01. Gene set enrichment analysis was performed for the DE genes with goseq (71) both for Gene Ontology (GO) (72, 73) terms and KEGG pathways (74, 75). The *P* values of the enrichment analyses were corrected for multiple testing, and a significance threshold of FDR < 0.05 was used to determine the terms and pathways with a significantly altered number of DE genes.

### Analysis of p53 and p21 regulation.

RIPC (3 times, 5 minutes each) was induced in mice; the kidneys were surgically removed after 4 hours. Kidneys were homogenized using the IKA T10 basis Homogenizer. Total RNA was extracted using the RNeasy Plus Mini kit (Qiagen) according to the manufacturer’s instructions. RNA was quantified using the NanoDrop 2000 Spectrophotometer (Thermo Fisher Scientific). In total, 0.5μg of RNA was then transcribed into cDNA using the RevertAid First Strand cDNA Synthesis Kit (Thermo Fisher Scientific). Transcript expression was analyzed in duplicate using the Bio-Rad CFX Connect. Primers were Designed with PrimerBlast (www.ncbi.nlm.nih.gov) with the following parameters: a maximum PCR product size of 200, a minimum intron length of 200, and a condition that primer pairs must be separated by at least 1 intron on the corresponding genomic DNA. Primer melting temperatures were set to a minimum temperature of 59°C, an optimum temperature of 62°C, and a maximum temperature of 65°C. The following primers were used: mus musculus Trp53 transformation related protein 53 (p53), transcript variant 1, mRNA: (For 5′ CCGAAGACTGGATGACTGCCA 3′, Rev 5′ TCAACATCCTGGGGCAGCAA 3′). Mus musculus cyclin-dependent kinase inhibitor 1A (p21), transcript variant 1, mRNA: (For 5′ CGCGGTGTCAGAGTCTAGGG 3′, Rev 5′ ACCGAAGAGACAACGGCACA 3′). Gene expression was normalized to the house keeping gene GAPDH (Mus musculus glyceraldehyde-3-phosphate dehydrogenase (Gapdh), transcript variant 1, mRNA: For 5′ GGCTCATGACCACAGTCCAT 3′, Rev 5′ GCCTGCTTCACCACCTTCT 3′) and then analyzed using the ΔΔCT method.

### Data and materials availability.

All data associated with this study are available in the main text or the supplementary materials. The transcriptomic data in this publication have been deposited in the National Center for Biotechnology Information Gene Expression Omnibus and are accessible through accession no. GSE206091 (https://www.ncbi.nlm.nih.gov/geo/query/acc.cgi?acc=GSE206091).

### Statistics.

Statistical analysis was performed with SPSS (version 22.0) using Mann-Whitney *U* (Wilcoxon) test or *t* test (2-tailed) as appropriate. More than 2 groups were compared using 1-way ANOVA followed by Bonferroni testing. Data distribution was assessed using Kolmogorov-Smirnov test or Shapiro-Wilks test. All data are represented as mean ± SEM. *P* < 0.05 was considered as statistically significant. For in vivo experiments, the provided *n* is the number of animals used per experiment. For in vitro experiments, *n* describes the number of independent experiments, each done at least in technical triplicates. To analyze the efficiency of RIPC (defined as [TIMP-2]*[IGFBP7] and HMGB1 elevation immediately after RIPC) for renal protection, we performed receiver operating characteristic (ROC) analyses and evaluated the AUC including 95% CI.

### Study approval.

All animal experiments were approved by the IRB (LANUV NRW). For studies with human patient material from the patient cohort of the RenalRIP trial (24), ethical approval from the IRBs at each participating study site site was granted, and written informed consent was obtained from the patients before inclusion into the study.

## Author contributions

JR and MM performed the experiments, analyzed the data, and wrote the manuscript in equal amounts and share first authorship based on the amount of contribution; KT, SM, ML, and JS performed experiments; TT analyzed the RNA-Seq data; JAK conceived of the mechanism and contributed to writing the manuscript; PR and HP contributed to writing the manuscript; and AZ conceived of the study, analyzed the data, and contributed to writing the manuscript.

## Supplementary Material

Supplemental data

## Version 1 06/21/2022

In-Press Preview

## Version 2 07/22/2022

Electronic publication

## References

[1] Kellum JA (2012). Kidney attack. JAMA.

[3] Singbartl K, Kellum JA (2012). AKI in the ICU: definition, epidemiology, risk stratification, and outcomes. Kidney Int.

[4] Chertow GM (2005). Acute kidney injury, mortality, length of stay, and costs in hospitalized patients. J Am Soc Nephrol.

[5] Basile DP (2012). Pathophysiology of acute kidney injury. Compr Physiol.

[6] Tan XH (2017). Fibroblast growth factor 2 protects against renal ischaemia/reperfusion injury by attenuating mitochondrial damage and proinflammatory signalling. J Cell Mol Med.

[7] He L (2014). Autophagy in acute kidney injury and repair. Nephron Clin Pract.

[8] Kaushal GP, Shah SV (2016). Autophagy in acute kidney injury. Kidney Int.

[9] Xu Y, Han J (2016). The necrosome in acute kidney injury. Semin Nephrol.

[10] Moonen L (2018). Epithelial cell cycle behaviour in the injured kidney. Int J Mol Sci.

[11] Wen X (2012). One dose of cyclosporine A is protective at initiation of folic acid-induced acute kidney injury in mice. Nephrol Dial Transplant.

[12] Price PM (2006). Dependence of cisplatin-induced cell death in vitro and in vivo on cyclin-dependent kinase 2. J Am Soc Nephrol.

[13] Megyesi J (1998). Induction of p21WAF1/CIP1/SDI1 in kidney tubule cells affects the course of cisplatin-induced acute renal failure. J Clin Invest.

[14] Price PM (2009). The cell cycle and acute kidney injury. Kidney Int.

[15] Boonstra J, Post JA (2004). Molecular events associated with reactive oxygen species and cell cycle progression in mammalian cells. Gene.

[16] Seo DW (2006). Shp-1 mediates the antiproliferative activity of tissue inhibitor of metalloproteinase-2 in human microvascular endothelial cells. J Biol Chem.

[17] Witzgall R (1994). Localization of proliferating cell nuclear antigen, vimentin, c-Fos, and clusterin in the postischemic kidney. Evidence for a heterogenous genetic response among nephron segments, and a large pool of mitotically active and dedifferentiated cells. J Clin Invest.

[18] Yang L (2010). Epithelial cell cycle arrest in G2/M mediates kidney fibrosis after injury. Nat Med.

[19] Gassanov N (2014). Remote ischemic preconditioning and renoprotection: from myth to a novel therapeutic option?. J Am Soc Nephrol.

[20] Hausenloy DJ (2007). Effect of remote ischaemic preconditioning on myocardial injury in patients undergoing coronary artery bypass graft surgery: a randomised controlled trial. Lancet.

[21] Hoole SP (2009). Cardiac remote ischemic preconditioning in coronary stenting (CRISP stent) study: a prospective, randomized control trial. Circulation.

[22] Kharbanda RK (2009). Translation of remote ischaemic preconditioning into clinical practice. Lancet.

[23] Thielmann M (2013). Cardioprotective and prognostic effects of remote ischaemic preconditioning in patients undergoing coronary artery bypass surgery: a single-centre randomised, double-blind, controlled trial. Lancet.

[24] Zarbock A (2015). Effect of remote ischemic preconditioning on kidney injury among high-risk patients undergoing cardiac surgery: a randomized clinical trial. JAMA.

[25] Zarbock A, Kellum JA (2016). Remote ischemic preconditioning and protection of the kidney--a novel therapeutic option. Crit Care Med.

[26] Er F (2012). Ischemic preconditioning for prevention of contrast medium-induced nephropathy: randomized pilot RenPro trial (renal protection trial). Circulation.

[27] Li C (2014). Limb remote ischemic preconditioning attenuates lung injury after pulmonary resection under propofol-remifentanil anesthesia: a randomized controlled study. Anesthesiology.

[28] Jensen HA (2011). Remote ischemic preconditioning protects the brain against injury after hypothermic circulatory arrest. Circulation.

[29] Meybohm P (2015). A multicenter trial of remote ischemic preconditioning for heart surgery. N Engl J Med.

[30] Hausenloy DJ (2015). Remote ischemic preconditioning and outcomes of cardiac surgery. N Engl J Med.

[31] Meersch M (2020). Effects of different doses of remote ischemic preconditioning on kidney damage among patients undergoing cardiac surgery: a single-center mechanistic randomized controlled trial. Crit Care Med.

[32] Mersmann J (2013). Attenuation of myocardial injury by HMGB1 blockade during ischemia/reperfusion is toll-like receptor 2-dependent. Mediators Inflamm.

[33] Bhattacharyya S (2018). Pharmacological inhibition of toll-like receptor-4 signaling by TAK242 prevents and induces regression of experimental organ fibrosis. Front Immunol.

[34] Wu H (2010). HMGB1 contributes to kidney ischemia reperfusion injury. J Am Soc Nephrol.

[35] Fan J (2007). Hemorrhagic shock induces NAD(P)H oxidase activation in neutrophils: role of HMGB1-TLR4 signaling. J Immunol.

[36] Huang X (2012). Prolonged early G(1) arrest by selective CDK4/CDK6 inhibition sensitizes myeloma cells to cytotoxic killing through cell cycle-coupled loss of IRF4. Blood.

[37] Legouis D (2015). Ex vivo analysis of renal proximal tubular cells. BMC Cell Biol.

[38] Hussey SE (2012). TAK-242, a small-molecule inhibitor of Toll-like receptor 4 signalling, unveils similarities and differences in lipopolysaccharide- and lipid-induced inflammation and insulin resistance in muscle cells. Biosci Rep.

[39] Guttridge DC (1999). NF-kappaB controls cell growth and differentiation through transcriptional regulation of cyclin D1. Mol Cell Biol.

[40] Alto LT, Terman JR (2017). Semaphorins and their signaling mechanisms. Methods Mol Biol.

[41] Jung JS (2019). Semaphorin-5B controls spiral ganglion neuron branch refinement during development. J Neurosci.

[42] Zager RA, Johnson ACM (2019). Acute kidney injury induces dramatic p21 upregulation via a novel, glucocorticoid-activated, pathway. Am J Physiol Renal Physiol.

[43] Roh JS, Sohn DH (2018). Damage-associated molecular patterns in inflammatory diseases. Immune Netw.

[44] Andersson U (2018). Extracellular HMGB1 as a therapeutic target in inflammatory diseases. Expert Opin Ther Targets.

[45] Wang H (2001). HMGB1 as a late mediator of lethal systemic inflammation. Am J Respir Crit Care Med.

[46] Le K (2018). Association of circulating blood HMGB1 levels with ischemic stroke: a systematic review and meta-analysis. Neurol Res.

[47] Lu CY (2012). Acute kidney injury: a conspiracy of Toll-like receptor 4 on endothelia, leukocytes, and tubules. Pediatr Nephrol.

[48] Meersch M (2014). Urinary TIMP-2 and IGFBP7 as early biomarkers of acute kidney injury and renal recovery following cardiac surgery. PLoS One.

[49] Yang QH (2009). Acute renal failure during sepsis: potential role of cell cycle regulation. J Infect.

[50] Wang Y (2017). Activation of the HMGB1‑TLR4‑NF‑κB pathway may occur in patients with atopic eczema. Mol Med Rep.

[51] Xia J, Worzfeld T (2016). Semaphorins and plexins in kidney disease. Nephron.

[52] Garcia S (2019). Role of semaphorins in immunopathologies and rheumatic diseases. Int J Mol Sci.

[53] Cuellar LM (2009). Identification and localization of novel genes preferentially expressed in human kidney glomerulus. Nephrology (Carlton).

[54] Liang B (2015). MiR-301a promotes cell proliferation by directly targeting TIMP2 in multiple myeloma. Int J Clin Exp Pathol.

[55] Shao X (2002). Epithelial-specific Cre/lox recombination in the developing kidney and genitourinary tract. J Am Soc Nephrol.

[56] Wang Z (2000). TIMP-2 is required for efficient activation of proMMP-2 in vivo. J Biol Chem.

[57] Matsuoka RL (2011). Class 5 transmembrane semaphorins control selective Mammalian retinal lamination and function. Neuron.

[58] Block H (2012). Crucial role of SLP-76 and ADAP for neutrophil recruitment in mouse kidney ischemia-reperfusion injury. J Exp Med.

[59] Li L (2010). IL-17 produced by neutrophils regulates IFN-gamma-mediated neutrophil migration in mouse kidney ischemia-reperfusion injury. J Clin Invest.

[60] Mocsai A (2006). Integrin signaling in neutrophils and macrophages uses adaptors containing immunoreceptor tyrosine-based activation motifs. Nat Immunol.

[61] Germena G (2015). Mutation in the CD45 inhibitory wedge modulates integrin activation and leukocyte recruitment during inflammation. J Immunol.

[62] Ewels P (2016). MultiQC: summarize analysis results for multiple tools and samples in a single report. Bioinformatics.

[63] Patro R (2017). Salmon provides fast and bias-aware quantification of transcript expression. Nat Methods.

[64] Frankish A (2019). GENCODE reference annotation for the human and mouse genomes. Nucleic Acids Res.

[65] Soneson C (2015). Differential analyses for RNA-seq: transcript-level estimates improve gene-level inferences. F1000Res.

[66] Zerbino DR (2018). Ensembl 2018. Nucleic Acids Res.

[67] Durinck S (2005). BioMart and Bioconductor: a powerful link between biological databases and microarray data analysis. Bioinformatics.

[68] Durinck S (2009). Mapping identifiers for the integration of genomic datasets with the R/Bioconductor package biomaRt. Nat Protoc.

[69] Love MI (2014). Moderated estimation of fold change and dispersion for RNA-seq data with DESeq2. Genome Biol.

[70] Zhu A (2019). Heavy-tailed prior distributions for sequence count data: removing the noise and preserving large differences. Bioinformatics.

[71] Young MD (2010). Gene ontology analysis for RNA-seq: accounting for selection bias. Genome Biol.

[72] Ashburner M (2000). Gene ontology: tool for the unification of biology. The Gene Ontology Consortium. Nat Genet.

[73] The Gene Ontology Consortium (2019). The Gene Ontology resource: 20 years and still GOing strong. Nucleic Acids Res.

[74] Kanehisa M, Goto S (2000). KEGG: kyoto encyclopedia of genes and genomes. Nucleic Acids Res.

[75] Kanehisa M (2019). New approach for understanding genome variations in KEGG. Nucleic Acids Res.

