# Supplementary material for: Remote ischemic preconditioning causes transient cell cycle arrest and renal protection by a NF-κB–dependent Sema5B pathway
Source: JCI Insight. 2022 Jul 22;7(14):e158523. doi: 10.1172/jci.insight.158523 (PMC9431690; doi:10.1172/jci.insight.158523)

# Supplemental Material

## Supplemental Figure Legends

Supplemental Figure 1: **Remote ischemic preconditioning (RIPC) performed on the upper leg of the front limb protects the kidney from IRI.** After induction of general anesthesia wildtype mice received either 3 cycles of 5 minute remote ischemic preconditioning by inflation of a blood pressure cuff (200 mmHg) positioned to upper leg of the front limb interrupted by 5 minute reperfusion intervals following cuff deflation. In the control group the cuff was just inflated to 20 mmHg, thus not resulting in limb ischemia. IRI was induced in wildtype mice by clamping of the renal pedicles for 32 minutes. 24 hours after the surgery, mice were sacrificed. Neutrophil accumulation in the kidney of mice who did receive **(A)** sham treatment or **(B)** RIPC was visualized by immunohistochemical staining of NE (exemplary images, scale bar equals 100µm, arrowheads indicate emigrated neutrophils). **(C)** The recruitment of neutrophils (PMNs) into the kidney was analyzed by flow cytometry (n=5). **(D)** Serum creatinine levels were measured by a photometric assay (n=5). **(E)** Plasma HMGB1 levels and **F)** urinary HMGB1 levels were analyzed before and after RIPC or control procedure (n=5). *=$p<0.05$

Supplemental Figure 2: **The protective effect of RIPC is not mediated via TLR5.** IRI was induced in WT control and TLR5$^{-/-}$ mice which did or did not receive RIPC prior to IRI induction by clamping of the renal pedicles for 32 minutes. 24 hours after the surgery, mice were sacrificed. **(A)** The recruitment of neutrophils (PMNs) into the kidney was analyzed by flow cytometry (n=4). **(B)** Serum creatinine levels were measured by a photometric based assay (n=4). The biomarkers TIMP2 and IGFBP7 were measured in urine samples **(C)** after RIPC application

and (D) 24 hours after IRI and renal tubular injury score **(E)** was assessed based on histology (n=4). *=p<0.05).

Supplemental Figure 3: **HMGB1 does not induce neutrophil TNFα release of ROS production.** **(A)** TNFα release from isolated WT neutrophils stimulated with vehicle, HMGB1 (100 ng/ml) or LPS control (n=5). **(B)** Superoxide production from HMBG1-treated (100 ng/mnl) and TNFα plus vehicle- or TNFα + HMBG1-treated (100 ng/ml) WT PMNs (n=4).

Supplemental Figure 4: **The induction of transient $G_0/G_1$ cell-cycle arrest is not mediated by RAGE or CXCR4.** **(A)** RT-PCR for Prominin-1, Aqp2 and CD31 was performed on lysates from isolated renal tubular epithelial cells. Gene expression was normalized to GAPDH expression and displayed relative to Prominin-1 (n=4). **(B)** Isolated murine renal tubular epithelial cells were treated with HMBG1 100ng/ml, HMBG1 100ng/ml plus a RAGE inhibitor (FPS-ZM1, 500nM) or HMBG1 100ng/ml plus a blocking CXCR4 antibody (clone 247506, R&D Systems, 10µg/ml) for 1 hour. The proportion of cells in $G_0/G_1$ phase was analyzed by measuring cellular DNA content by flow cytometry (n=6).

Supplemental Figure 5: **RIPC ameliorates glycerol-induced AKI.** After induction of general anesthesia renal IRI was induced in WT control mice by intramuscular injection of glycerol. 18 hours after the procedure, mice were sacrificed. **(A)** The recruitment of neutrophils (PMNs) into the kidney was analyzed by flow cytometry (n=4). **(B)** Serum creatinine levels were measured by a photometric based assay (n=4). *=p<0.05.

Supplemental Figure 6: **RIPC upregulates p53 and p21 expression.** After induction of general anesthesia RIPC (3x5 min) or sham procedure was performed in WT mice. After 4 hours the mice were sacrificed, the kidneys were surgically removed and primary TECs were isolated from the kidney. The expression of p53 and p21 were analyzed by qRT-PCR (n=6). *=$p<0.05$.

Supplemental Figure 7: **Individual TIMP-2 and IGFBP7 values for TIMP2*IGFBP7 compound values presented in the main figure file.** Individual values for **(A)** TIMP-2 and **(B)** IGFBP7 from Figure 3E. Individual values for **(C)** TIMP-2 and **(D)** IGFBP7 from Figure 3F. Individual values for **(E)** TIMP-2 and **(F)** IGFBP7 from Figure 4C. Individual values for **(G)** TIMP-2 and **(H)** IGFBP7 from Figure 6G. Individual values for **(I)** TIMP-2 and **(J)** IGFBP7 from Figure 8C. (n=4). *=$p<0.05$.

# Supplemental Figure 1

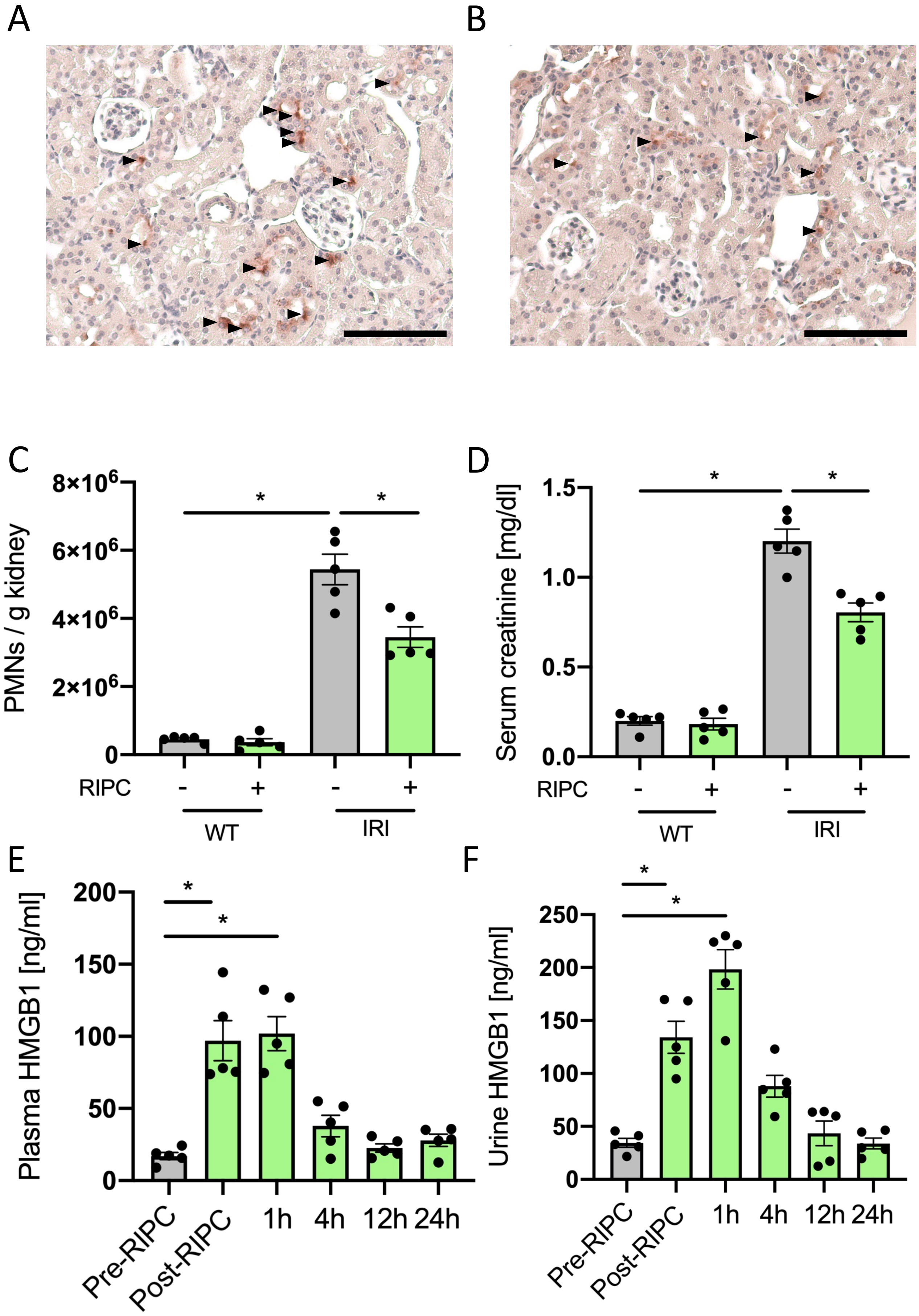

# Supplemental Figure 2

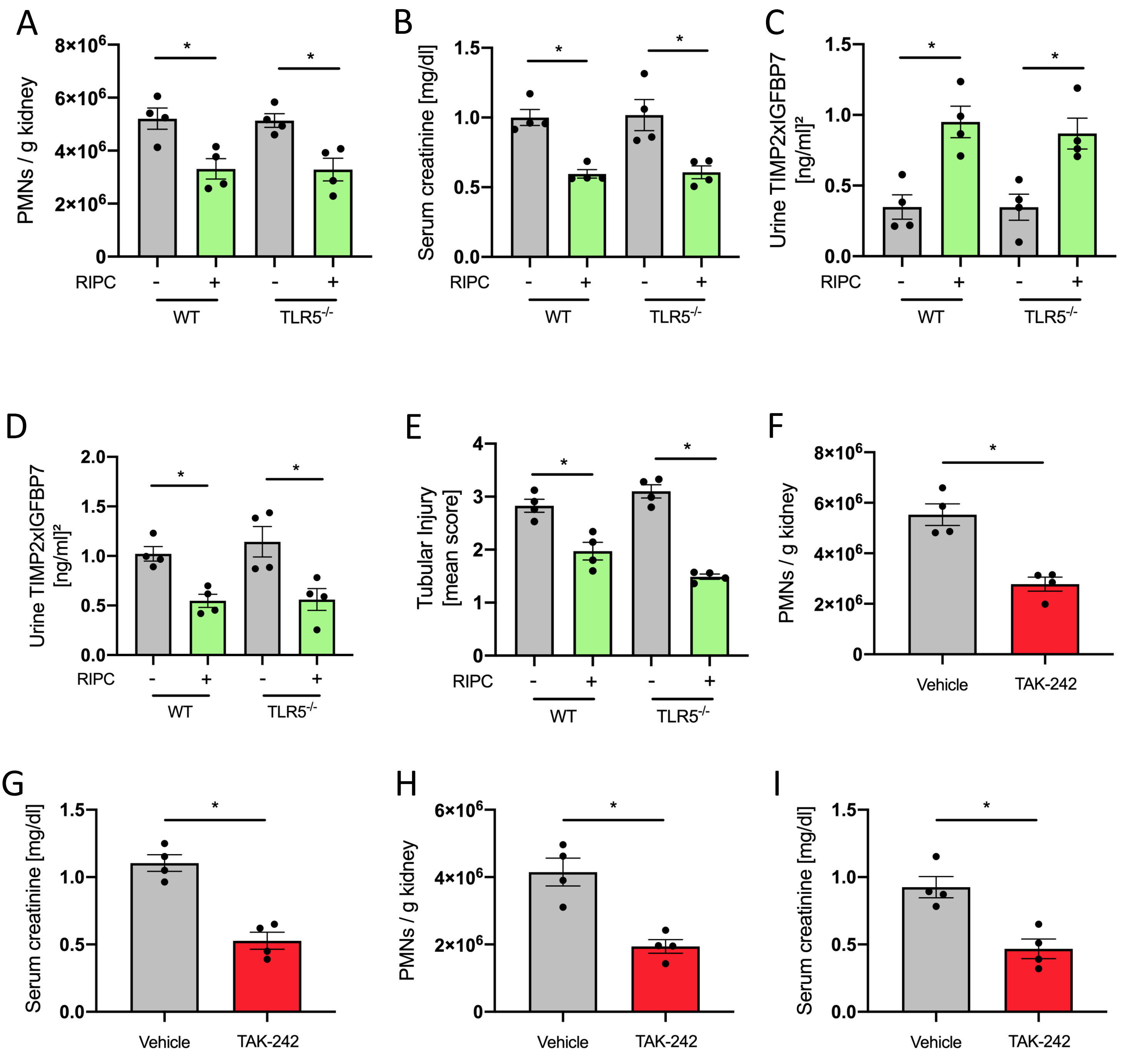

# Supplemental Figure 3

A

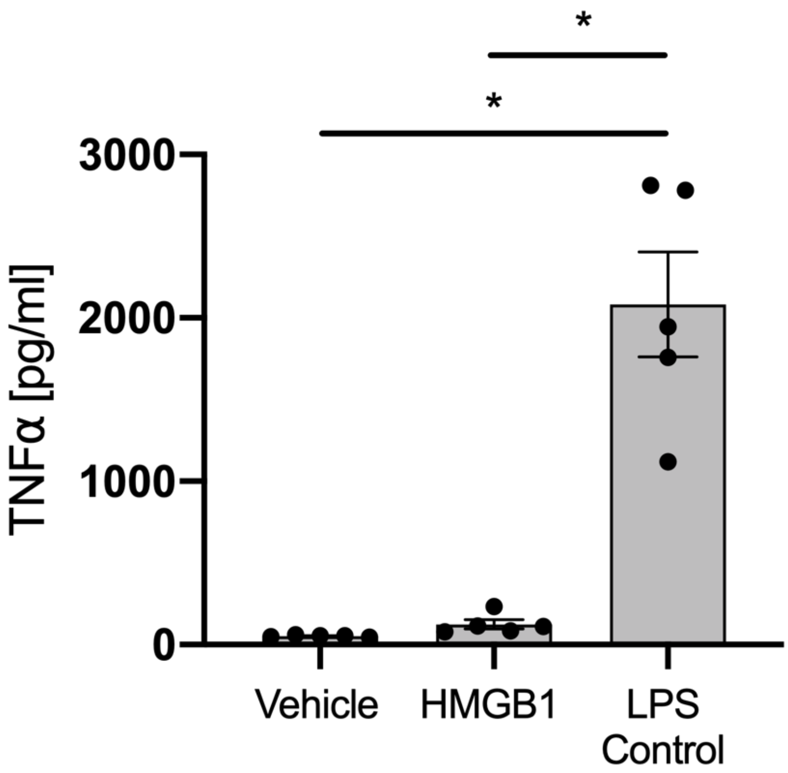


B

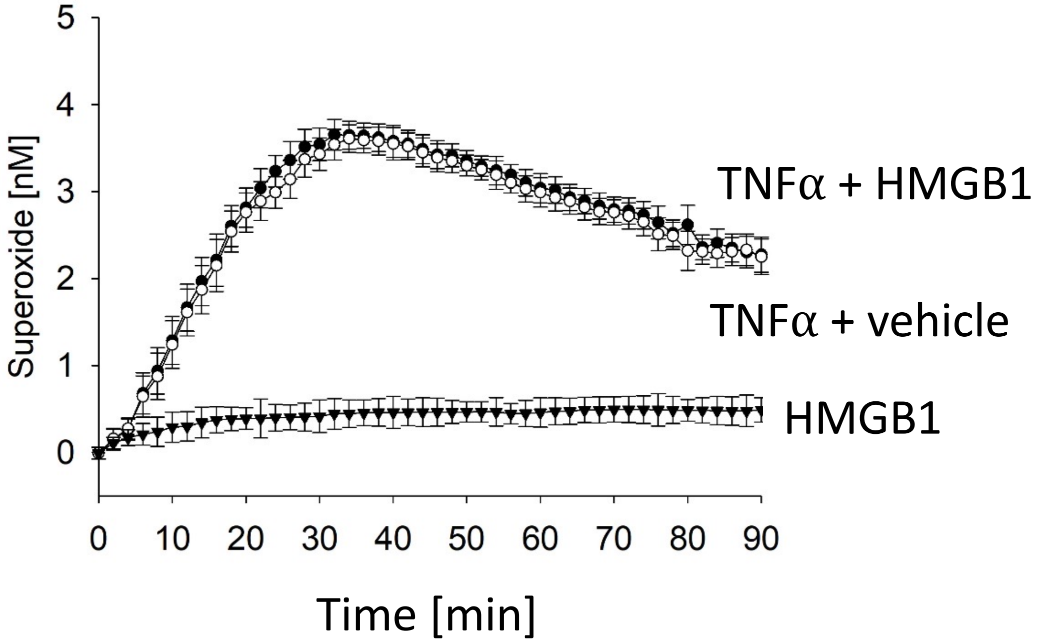

# Supplemental Figure 4

A

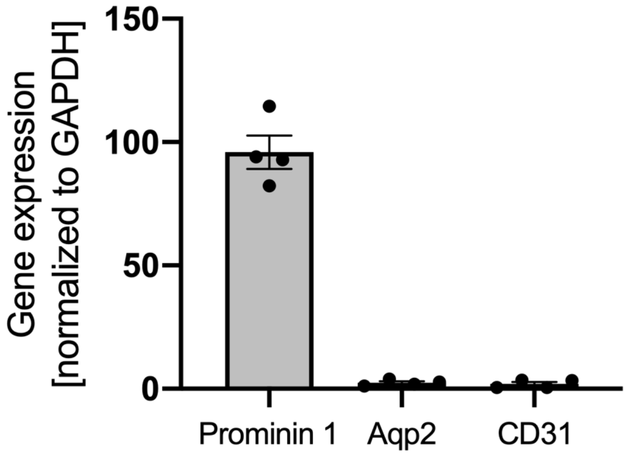


B

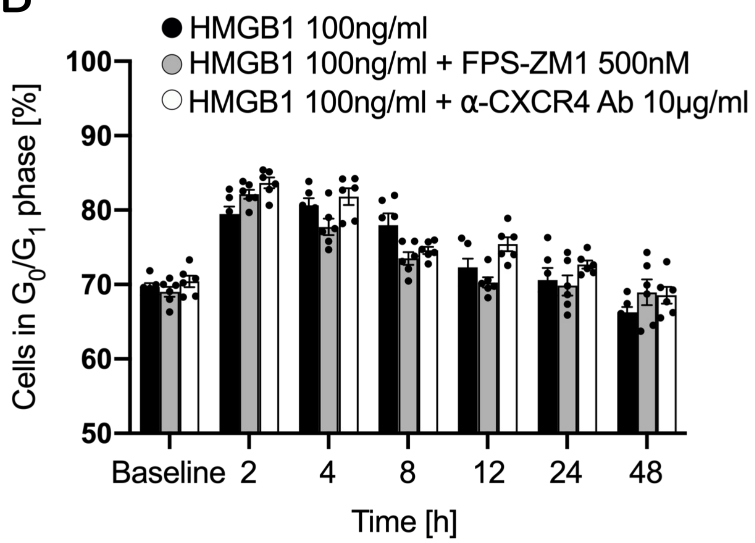

# Supplemental Figure 5

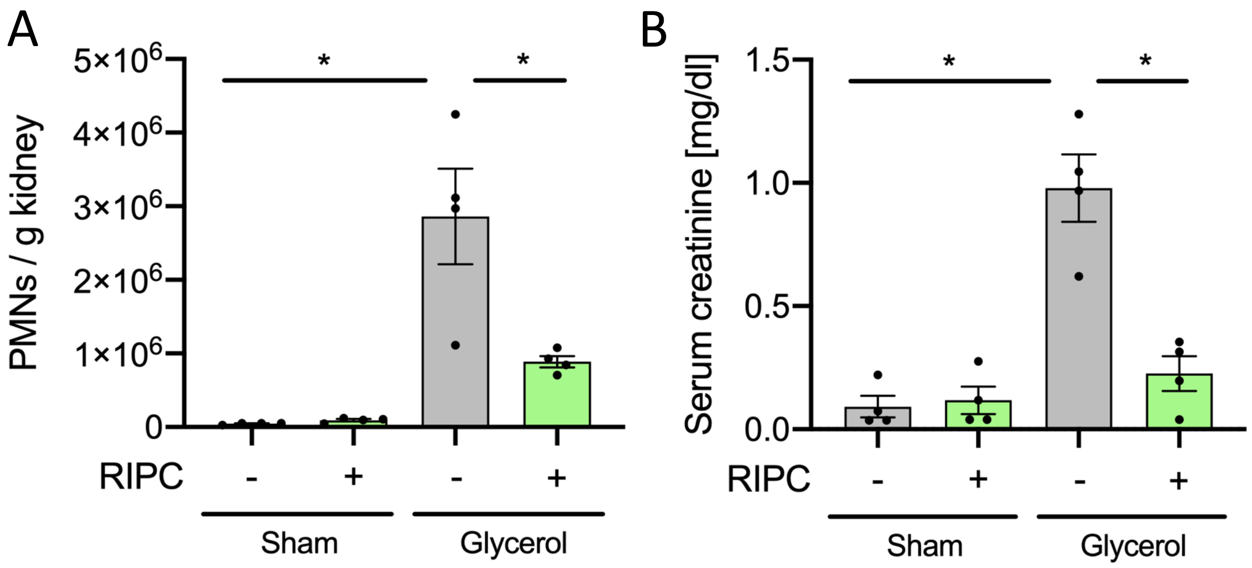

# Supplemental Figure 6

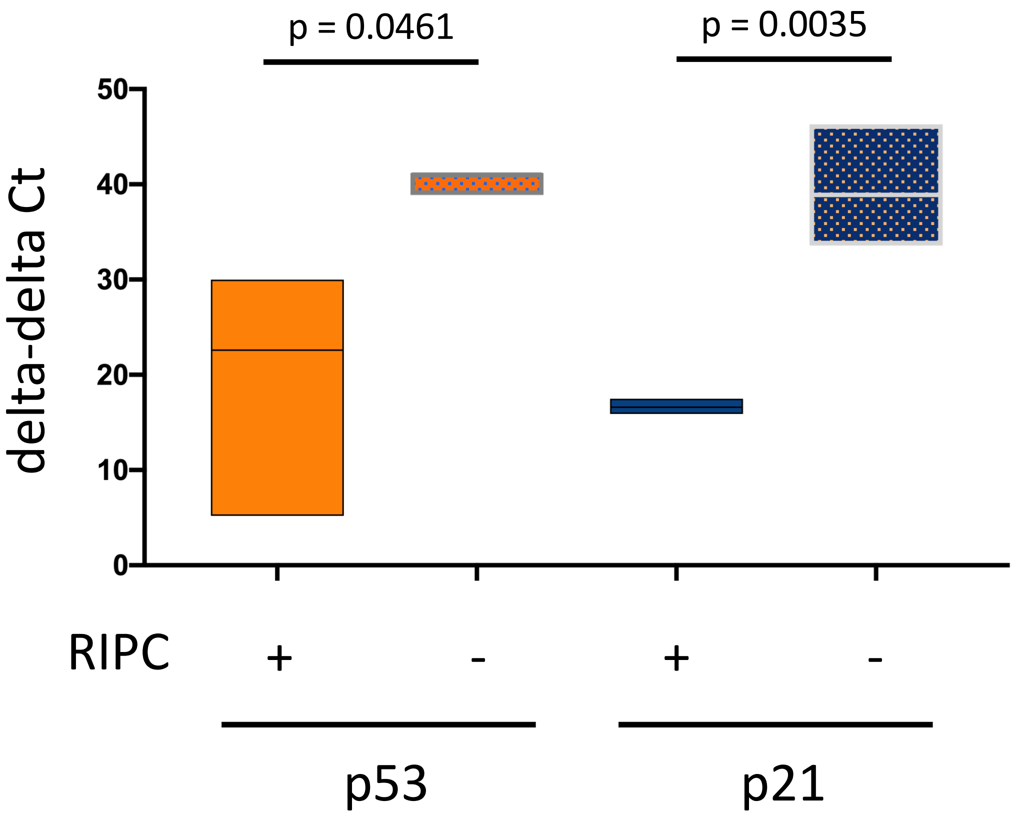

# Supplemental Figure 7

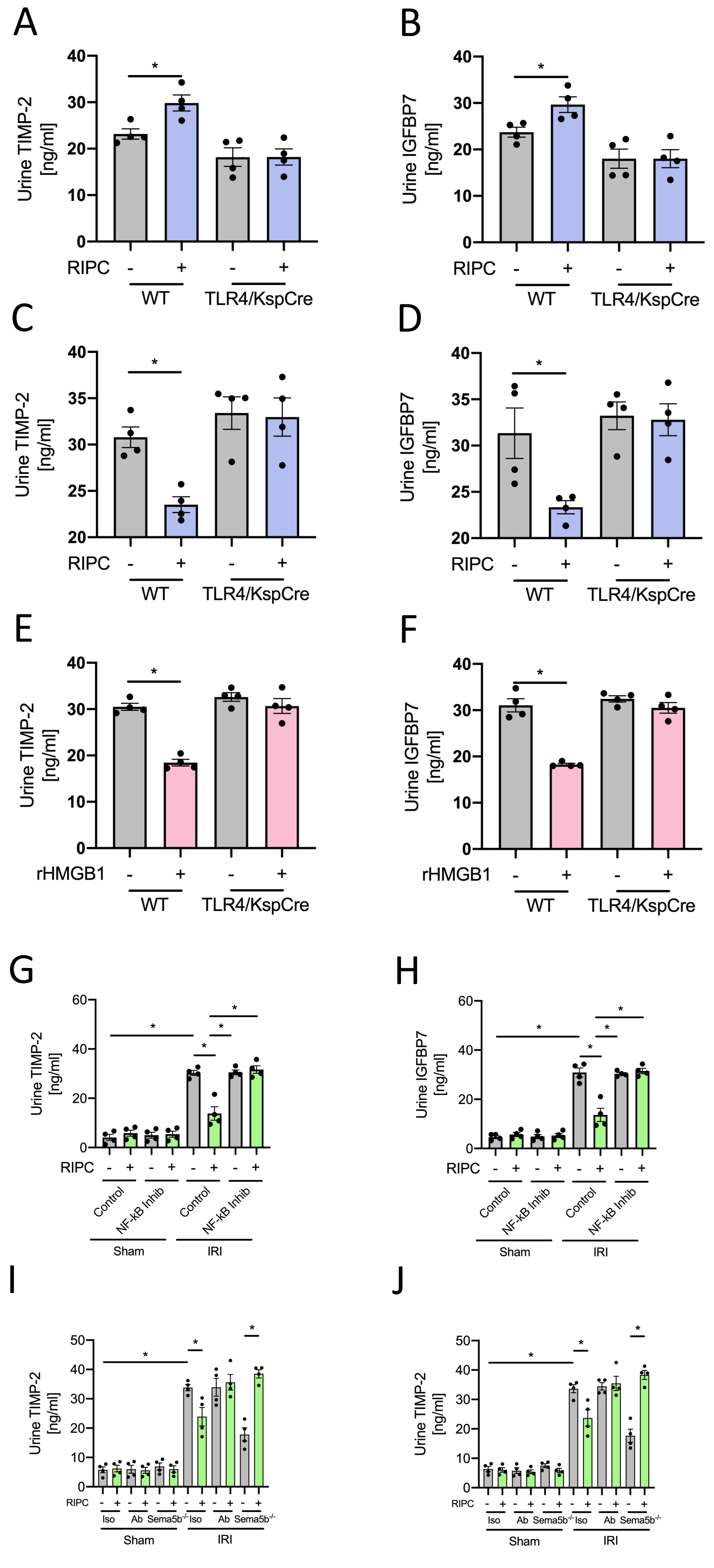

Supplement: Supplemental data [file jciinsight-7-158523-s074.pdf]
